# Autophagy and Endoplasmic Reticulum Stress during Onset and Progression of Arrhythmogenic Cardiomyopathy

**DOI:** 10.3390/cells11010096

**Published:** 2021-12-29

**Authors:** Mark Pitsch, Sebastian Kant, Corinna Mytzka, Rudolf E. Leube, Claudia A. Krusche

**Affiliations:** Institute of Molecular and Cellular Anatomy, RWTH Aachen University, Wendlingweg 2, 52074 Aachen, Germany; mpitsch@ukaachen.de (M.P.); skant@ukaachen.de (S.K.); corinna.mytzka@rwth-aachen.de (C.M.)

**Keywords:** ARVC, arrhythmogenic cardiomyopathy, desmoglein, autophagy, ER stress, Chop

## Abstract

Arrhythmogenic cardiomyopathy (AC) is a heritable, potentially lethal disease without a causal therapy. AC is characterized by focal cardiomyocyte death followed by inflammation and progressive formation of connective tissue. The pathomechanisms leading to structural disease onset and progression, however, are not fully elucidated. Recent studies revealed that dysregulation of autophagy and endoplasmic/sarcoplasmic reticulum (ER/SR) stress plays an important role in cardiac pathophysiology. We therefore examined the temporal and spatial expression patterns of autophagy and ER/SR stress indicators in murine AC models by qRT-PCR, immunohistochemistry, in situ hybridization and electron microscopy. Cardiomyocytes overexpressing the autophagy markers LC3 and SQSTM1/p62 and containing prominent autophagic vacuoles were detected next to regions of inflammation and fibrosis during onset and chronic disease progression. mRNAs of the ER stress markers Chop and sXbp1 were elevated in both ventricles at disease onset. During chronic disease progression Chop mRNA was upregulated in right ventricles. In addition, reduced Ryr2 mRNA expression together with often drastically enlarged ER/SR cisternae further indicated SR dysfunction during this disease phase. Our observations support the hypothesis that locally altered autophagy and enhanced ER/SR stress play a role in AC pathogenesis both at the onset and during chronic progression.

## 1. Introduction

Arrhythmogenic cardiomyopathy (AC) is a frequent cause of sudden cardiac death in young adults and may lead to heart failure [1,2]. It is characterized by focal fibrofatty replacement of myocardium leading to ventricular dilation and ventricular wall aneurysms [3,4]. The term arrhythmogenic right ventricular cardiomyopathy/dysplasia (ARVC/D) has been widely used because of the right ventricular dominance of the pathologies, but reports of significant left ventricular and bi-ventricular involvement have promoted the use of the more general term AC [5,6]. Disease prevalence has been reported to be between 1:2000 [7] and 1:5000 [1]. AC is regarded as a disease of the desmosome since most known causative mutations affect genes encoding proteins of the desmosomal complex [8]. Human AC progression starts after a preclinical asymptomatic phase with the onset of cardiac arrhythmias. Since potentially lethal arrhythmias may occur early in the disease without prior warning symptoms, early diagnosis of AC is of utmost importance [4,9]. After the appearance of electrical instability multiple stages of progressive pathological myocardial remodeling may eventually lead to heart failure [10]. The interval between the first ECG changes and symptoms of right and/or left heart failure, however, is highly variable and the mechanisms contributing to the manifestation of heart failure are still underinvestigated.

Among potential pathomechanisms contributing to heart disease, autophagy and ER/SR stress have gained much attention in recent years [11,12,13]. Autophagy is induced by multiple physiological and pathological stimuli, e.g., hormones, developmental processes, starvation, hypoxia, accumulation of dysfunctional organelles and unfolded/misfolded proteins in the ER. It serves to remove damaged and superfluous organelles and proteins to re-establish cellular homeostasis [14,15,16]. The process of macroautophagy, hereafter referred to as autophagy, is characterized by the formation of intracellular autophagosomes, which are surrounded by two membranes. Fusion of autophagosomes with lysosomes leads to the formation of autolysosomes. They degrade the engulfed organelles and cytoplasmic components. The resulting breakdown products are released into the cytoplasm for reuse. This multi-step process is guided by a complex molecular machinery including the protein LC3 for autophagosome formation and SQSMT1/p62 for guiding substrates into autophagosomes for degradation [17]. While autophagy is essential for regular development, tissue maturation and restructuring by providing a physiological cell-protective stress response, enhanced or reduced autophagy have been shown to play important roles in diseases of various organs [18,19,20]. This is also true for multiple cardiovascular disorders, in which impaired autophagy has been described in combination with ER/SR stress [21,22,23]. A detailed study on the role of autophagy and ER/SR stress in AC onset and progression, however, is still lacking. The goal of the current project was to fill this knowledge gap. Our motivation was further stimulated by the occasional observation of an increased number of autophagosomes and dilation of ER/SR cisternae and T-tubules in cardiomyocytes of murine AC models [24,25]. We therefore decided to systematically analyze the temporal and spatial expression of marker proteins and ultrastructural signs of autophagy and ER stress in the hearts of established *Dsg2*-mutant mouse lines [24,26,27,28]. The *Dsg2* gene encodes the desmosomal cadherin desmoglein 2, which has been linked to AC in human patients [29,30,31]. The constitutive *Dsg2*-mutant (*Dsg2^MT^*) and cardiomyocyte-specific *Dsg2*-knock out (*Dsg2^cKO^*) mouse lines develop an AC-like phenotype. They display key features of AC progression that are also observed in human AC patients [10,24,26,27,32].

In the present study, we provide strong evidence for an early involvement of autophagy and ER/SR stress in AC initiation, manifestation and disease progression.

## 2. Material and Methods

### 2.1. Animals

We used two mouse strains that have different genetic alterations of the *Dsg2* gene and develop AC-like cardiac alterations [24,26,27,28,33]: (i) *Dsg2*-mutant (*Dsg2^MT^*) mice lack exons 4–6, which encode major parts of the extracellular EC1-EC2 domains of the DSG2 protein. Homozygous *Dsg2^MT^* mice were compared with *Dsg2* wild-type mice. (ii) The *Dsg2^cKO^* strain contains cardiomyocyte-specific ablation of exons 4–5 of the *Dsg2* gene through Myh6-Cre-mediated recombination of the floxed *Dsg2^flox(E4−5)^* allele. Myocardial samples from homozygous *Dsg2^cKO^* mice were compared to those of healthy litter mates, which lacked the Cre-recombinase and contained only the *Dsg2^flox(E4−5)^* alleles. We analyzed mice in groups at the age of 2 weeks (range 2–2.5 weeks), 4 weeks, 6 weeks (range 5–6 weeks), 12 weeks (range 11–13 weeks), 30 weeks (range 22–38 weeks) and 1 year (range 50–66 weeks).

Animals were housed in the central animal facility of the Uniklinik RWTH Aachen University. They received a standard rodent lab diet (Ssniff, Soest, Germany) and had free access to food and water. All animal experiments were conducted in accordance with the guidelines for the care and use of laboratory animals and were approved by the Landesamt für Natur, Umwelt und Verbraucherschutz Nordrhein-Westfalen (LANUV; reference number 84-02.04.2015.A190 and approvals according to §4 of the German Animal Welfare Act). At necropsy, hearts were visually examined, excised and prepared for subsequent analyses.

### 2.2. Electron Microscopy

Cardiac ultrastructure was analyzed in homozygous *Dsg2^MT^* and healthy wild-type (*Dsg2^WT^*) mice at the age of 2 weeks (N = 8 for *Dsg2^MT^*, N = 3 for *Dsg2^WT^* and N = 1 for *Dsg2^mt/wt^* mice), 3 weeks (N = 1 for *Dsg2^MT^* and N = 1 for *Dsg2^mt/wt^* mice), 12 and 30 weeks (N = 4 for *Dsg2^MT^* and N = 2 for *Dsg2^WT^* per time point). After cervical dislocation, hearts were retrogradely perfused with relaxation buffer (30 mM KCl, 5% glucose). Right and left ventricles were thereafter separated, placed in Trump’s fixative (3.7% formaldehyde, 1% glutaraldehyde, in 85 mM phosphate buffer) and cut into pieces of 1 mm^3^. After incubation in fresh fixative for 2 h and in 1% OsO_4_ for 1 h, samples were incubated in 0.5% uranyl acetate buffered in 50 mM sodium maleate (pH 5.2) for 2 h in the dark. After dehydration, samples were embedded in araldite using acetone as intermedium. The final araldite polymerization was initiated by the addition of 2% DMP-30 and continued for 48 h at 60 °C. Ultra-thin sections of 70 nm were prepared on a microtome. To enhance contrast, sections were treated with 3% aqueous uranyl acetate buffer for 4 min in the dark and then with 0.08 M lead citrate for 3 min. Images were acquired using an EM10 (Carl Zeiss Microscopy GmbH, Jena, Germany) equipped with a digital camera using the iTEM software (both from Olympus, Münster, Germany).

### 2.3. Paraffin Embedding, Sectioning and Histological Staining

Hearts were excised, rinsed in phosphate buffered saline (PBS) and fixed in 4% (*w*/*v*) neutrally buffered formaldehyde overnight. After dehydration in an ascending series of isopropanol, tissue samples were immersed in paraffin at 60 °C overnight with one change of paraffin. Paraffin blocks were cut on a rotary microtome (Thermo Fisher Scientific Inc., Waltham, MA, USA). The 5 µm-thick sections were transferred to SuperFrost Plus microscope slides (Thermo Fisher Scientific) and attachment was ensured by incubating sections on a heat plate at 37 °C overnight.

To assess the histomorphology of control and diseased hearts two standard histological staining methods were used. Heidenhain’s AZAN trichrome stain was applied to visualize fibrotic changes of the diseased myocardium. Hematoxylin-eosin staining was used to study immune cell infiltrates and cardiomyocyte morphology.

### 2.4. LC3 and Cleaved Caspase 3 (CC3) Immunohistochemistry

For the detection of LC3 in paraffin-embedded heart sections, tissue samples from 2, 4, 6, 12 and 30 week-old *Dsg2^cKO^* mice were examined (N = 4–6). Age-matched homozygous *Dsg2^flox(E4−5)^* mice served as controls (N = 3–4 except for 2 week-old mice N = 7). Cleaved caspase 3 was analyzed in sections of 18 day-old *Dsg2^cKO^* and 4 and 30 week-old *Dsg2^MT^* mice (N = 3–5) and the respective age matched *Dsg2^flox(E4−5)^* or *Dsg2^WT^/Dsg2^wt/mt^* controls (N = 3–5). Sections were deparaffinized in xylene and rehydrated in a descending ethanol series. An incubation step in 3% H_2_O_2_/70% ethanol for 10 min in the dark was included to ensure blockage of endogenous peroxidase. In case of LC3 epitope retrieval was achieved by heating the slides in 10 mM citrate buffer (pH 6.0) for 30 min at 94 °C in a water bath. CC3 epitope retrieval was achieved by heating sections in 1 mM EDTA buffer (pH 8) for 10 min at 100 °C. After cooling down to room temperature, the blocking solution provided in the ZytoChem-Plus HRP-Polymer-Kit (Zytomed Systems GmbH) was applied for 5 min to prevent unspecific antibody binding. Next, sections were subjected to the primary rabbit anti-LC3 antibody (GTX127375; GeneTex Inc., Irvine, CA, USA) diluted 1:500 in PBS for 60 min at room temperature or the primary rabbit anti-CC3 antibody (#9661; Cell Signaling Technology, Danvers, Massachusetts, USA) diluted 1:300 in PBS for an overnight incubation at 4 °C. After two washing steps of 5 min (Tris wash buffer: 50 mM Tris/HCl pH 7.5; 0.3 M NaCl, 0.045% Tween 20), sections were incubated with the horseradish peroxidase (HRP)-coupled polymer from the ZytoChem-Plus HRP-Polymer-Kit for 30 min. For color development, the DAB Quanto System (Thermo Fisher Scientific) was applied for 4.5 min or 6 min. Following counterstaining with hematoxylin (Carl Roth GmbH, Karlsruhe, Germany or Zytomed Systems, Berlin, Germany), slides were mounted and images were acquired using an Axiophot microscope equipped with an Axiocam Cc5 camera (both from Zeiss, Oberkochen, Germany). Negative controls were obtained either by omitting the primary antibody or by using non-IgG rabbit normal serum instead of the primary antibody. Murine brain or lymph nodes served as a positive control tissue.

### 2.5. SQSTM1/p62 Immunofluorescence

For immunofluorescence staining of SQSTM1/p62, hearts of 2, 4, 6 and 30 week-old and 1 year-old *Dsg2^cKO^* mice (N = 4–6 per age group, except for 2 weeks [N = 1]) and age-matched homozygous *Dsg2^flox(E4−5)^* mice (N = 3–5 per age group, except for 2 weeks [N = 2]) were dissected, covered in Tissue-Tek^®^ O.C.T. Compound (Science Services, Munich, Germany) and snap-frozen in liquid nitrogen. In case of storage for later use, samples were kept at −40 °C. Eight µm thick cryosections were prepared using a cryostat at −20 °C and mounted on SuperFrost Plus microscope slides (Thermo Fisher Scientific). After drying at room temperature, sections were fixed in acetone precooled to −20 °C for 10 min. Blocking was performed by a 30 min incubation in blocking buffer (2.5% BSA in PBS) containing 2% normal goat serum. Sections were then covered with the primary guinea pig anti-p62 IgG antibody (GP62-C; PROGEN Biotechnik GmbH, Heidelberg, Germany) diluted 1:100 in blocking buffer and incubated at 4 °C overnight. After subsequent washing, the secondary goat anti-guinea pig IgG antibody conjugated with Alexa Fluor 555 (A21435; Invitrogen AG, Carlsbad, CA, USA) diluted 1:500 in blocking buffer was applied for 60 min at room temperature. 0.1% Sudan Black B (Merck KGaA, Darmstadt, Germany) dissolved in 70% ethanol was used for 30 min to quench autofluorescence and, after washing, nuclei were counterstained with 2 µg/mL Hoechst 33,342 (Thermo Fisher Scientific) in PBS for 30 min in the dark. Sections were mounted with Mowiol^®^ 4–88 (Merck, Darmstadt, Germany). Images were acquired using an ApoTome.2 microscope setup (Zeiss). Negative controls were prepared by omitting the primary antibody or by replacing the first antibody with guinea pig serum (1:100) in 2.5% BSA-containing PBS.

### 2.6. Real-Time PCR

For measuring mRNA expression, hearts of *Dsg2^cKO^*, *Dsg2^MT^*, *Dsg2^flox(E4−5)^* control and wild-type mice were excised and rinsed in PBS. We analyzed ventricle-specific gene expression at the age of 2, 4, 12 and 30 weeks (N = 4–7 animals per genotype and age). After separating left and right ventricles, tissue samples were homogenized with the help of a Dounce homogenizer in RNA lysis buffer supplied with the peqGOLD Total RNA Kit (VWR International). Using this kit and the peqGOLD DNase I Digest Kit (VWR International), total RNA was isolated according to the instructions of the manufacturer. cDNA was prepared by reverse-transcription of total mRNA using the Transcriptor First Strand cDNA Synthesis Kit with oligo-(dT)_18_ primer (both from Roche). Primers for qRT-PCRs were designed and ordered using the Universal Probe Library System Assay Design Software (Roche). Primer efficiencies were measured according to Pfaffl [34]. FastStart Essential DNA Probes Master Kit (Roche) and cDNA were added to combinations of primers and corresponding UPL probes according to Table 1. qRT-PCRs were conducted using a LightCycler^®^ 96 (Roche) with each sample setup in duplicate. Expression of hydroxymethylbilane synthase (Hmbs) as a housekeeping gene and the experimentally determined primer pair efficiencies were used for relative quantification of target mRNA expression using the LightCycler^®^ 96 software (Roche).

### 2.7. In Situ Hybridization

Sqstm1/p62 and Chop mRNAs were localized in 5 µm-thick sections of paraffin-embedded hearts using the ViewRNA ISH Tissue 1-Plex Assay Kit and the ViewRNA™ Chromogenic Signal Amplification Kit (QVT0050 and QVT0200, respectively; Thermo Fisher Scientific). *Dsg2^cKO^*, *Dsg2^MT^* and the respective control mice were studied at the age of 4 and 30 weeks and 1 year (N = 3–4 for *Dsg2^MT^/Dsg2^cKO^* and N = 1–2 for *Dsg2^WT^/Dsg2^flox(E4−5)^*). Sections were immersed in pretreatment solution and heated to 95 °C for 10 min followed by protease digestion at 40 °C for 20 min. ViewRNA Type 1 Probe Sets were used for detection of p62 mRNA (VB1–20466), Chop mRNA (VB1–17717) and actin alpha cardiac muscle 1 (Actc1)-mRNA (VB1–13263) as positive control. Nuclear counterstaining was done with hematoxylin (Morphisto GmbH, Frankfurt am Main, Germany) and with Hoechst 33,342 (Thermo Fisher Scientific). Sections were mounted with Mowiol^®^ 4–88 (Merck). Images were acquired using an ApoTome.2 microscope setup (Zeiss). Negative controls were prepared by replacing the specific probe with Probe Set Diluent QF buffer.

### 2.8. Statistical Methods

Statistical analyses were performed with GraphPad Prism5 (GraphPad Software Inc., San Diego, CA, USA). If not stated differently, results are presented as mean value ± standard deviation (SD). The two-tailed Mann-Whitney-Test was used to analyze statistical significance since animal numbers were too low to apply a normality test in some age groups. *p* < 0.05 was considered as statistically significant: * (*p* < 0.05), ** (*p* < 0.01), *** (*p* < 0.001).

## 3. Results

Morphological manifestation of the AC phenotype occurs at the age of 14 ± 1–2 days in *Dsg2^MT^* mice and slightly later, i.e.**,** at 18 ± 1–2 days in *Dsg2^cKO^* mice (Figure S1). Disease onset is characterized by focal cardiomyocyte necrosis (see Figure S2a,d,g,h,j,k and [28]). The size of the myocardial areas affected by cardiomyocyte necrosis shows considerable inter-individual differences (compare Figure S2a,d). The time line of subsequent pathogenesis is identical in both *Dsg2*-mutant mouse lines (Figures S1–S3). Initial cardiomyocyte necrosis is followed by an aseptic inflammatory response and replacement fibrosis during the acute disease phase, which is completed by the age of 12 weeks [27,28,33]. Similar sequels have been described in other *Dsg2*-based mouse models [35,36,37]. We regularly observe the formation of different types of fibrosis after disease onset in *Dsg2^MT^* and *Dsg2^cKO^* mice (Figures S2b,c,e,f and S3). Increasing amounts of collagen fibers are indicative of scar maturation, which allows to distinguish between an early (no collagen fibrils), immature (few collagen fibrils, many interstitial cells) and mature state (dense collagen fibrils and few interstitial cells; Figure S2a–f). We estimate that it takes about 4–5 weeks to establish a mature scar in our AC mouse models. It is furthermore possible to distinguish lesions consisting of loose or dense connective tissue and lesions containing calcified cardiomyocytic remnants embedded in a dense collagenous network (Figures S2b,c,e,f and S3b–f). All scar types are found in right and left ventricles and the septum. The chronic AC phase starts at the age of 12 weeks and may lead to heart failure or sudden cardiac death. It is characterized by a slow but continuous loss of vital cardiomyocytes and is accompanied by persisting low level immune cell infiltration, chamber dilation and an increase of interstitial fibrosis (Figures S2c,f and S3f [27,28,33,36]). In addition, pathological cardiomyocyte hypertrophy was observed during chronic disease progression [26].

### 3.1. LC3-Positive Autophagosomes Accumulate in Cardiomyocytes of Dsg2-Mutant Mice

**Figure 1:**
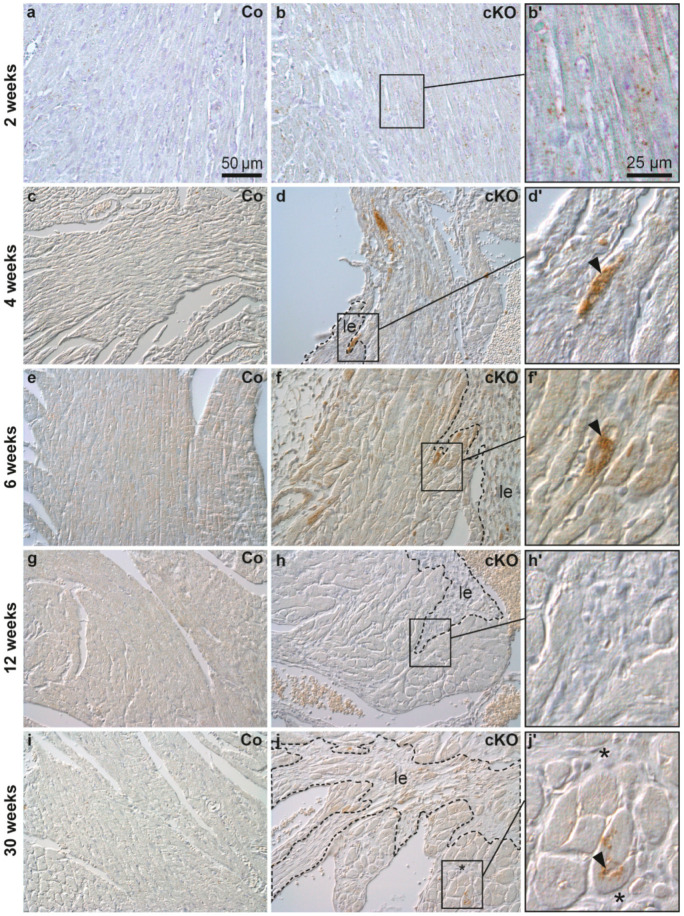
LC3-positive cardiomyocytes are detected near fibrotic lesions during structural disease onset and disease progression in *Dsg2* knock-out hearts. The figure shows representative micrographs taken from left ventricles. Myocardial LC3 staining is depicted using interference contrast microscopy. Left column: homozygous *Dsg2^flox(E4−5)^* control mice (Co; **a,c,e,g,i**); middle and right columns: *Dsg2^cKO^* mice (cKO). At the age of 2 weeks, circularly arranged cardiomyocytes of *Dsg2^cKO^* mice present more LC3-positive granular dots than those of the wild type. The LC3-positive vacuoles are distributed throughout the cell body (**b**,**b′**). At 4–6 weeks, cardiomyocytes located in close proximity to the forming scar tissue (demarcated with a dotted line, le) contain densely-packed LC3-positive granules (arrowheads; (**d**,**d′**,**f**,**f′**)). At 12 weeks, hardly any LC3-positive granules are detectable in *Dsg2^cKO^* cardiomyocytes (**h**,**h′**), but at 30 weeks LC3-positive granules are clearly present in cardiomyocytes lining interstitial (* in (**j**,**j′**)) or replacement fibrosis (le, marked by dotted lines in (**j**)). Scale bars: 50 µm in a (same magnification in entire left and middle column), 25 µm in (**b′**) (same magnification in entire right column).

To find out whether autophagosome formation takes part in AC pathogenesis, LC3 expression was assessed by immunohistochemistry in *Dsg2^cKO^* hearts during the different disease phases (Figure 1). Induction of autophagy elicits binding of LC3 to the inner and outer autophagosomal membrane. Detection of LC3 is therefore a well-established way to visualize autophagosomes [38,39,40]. A few LC3-positive granules were found in the maturing myocardium of 2 week-old *Dsg2^flox^* control hearts but were rare in older control animals (Figure 1c,e,g,i). In contrast, LC3 immunohistochemistry revealed cardiomyocytes with conspicuous granular staining primarily in the circular muscle layers of 2 week-old *Dsg2^cKO^* hearts, in which we detected no lesion (Figure 1b,b′). In *Dsg2^cKO^* hearts with incipient lesions LC3-positive cardiomyocytes were detected in healthy-appearing cardiomyocytes that were often located in the vicinity of areas containing necrotic cardiomyocytes and LC3-positive immune cells (Figure S4a–c). At the age of 4 and 6 weeks, *Dsg2^cKO^* mice presented individual cardiomyocytes with excessive LC3-positive vacuoles. These cells localized next to maturing fibrotic scars (Figure 1d,d′,f,f′). LC3-positive cardiomyocytes were not detectable anymore at the end of the acute disease phase in 12 week-old *Dsg2^cKO^* mice (Figure 1h,h’), i.e., at a time, when the fibrotic scars had matured and the immune reaction had decayed [28,36]. Surprisingly, LC3-positive granules and structures reappeared in cardiomyocytes during the chronic disease phase in 30 week-old *Dsg2^cKO^* mice (Figure 1j,j′), albeit to a far lesser extent than at the beginning of the acute disease phase.

### 3.2. Increased SQSTM1/p62 Protein and Sqstm1/p62 mRNA Levels Are Detected during the Acute Phase and Late Progression of Murine Arrhythmogenic Cardiomyopathy

**Figure 2:**
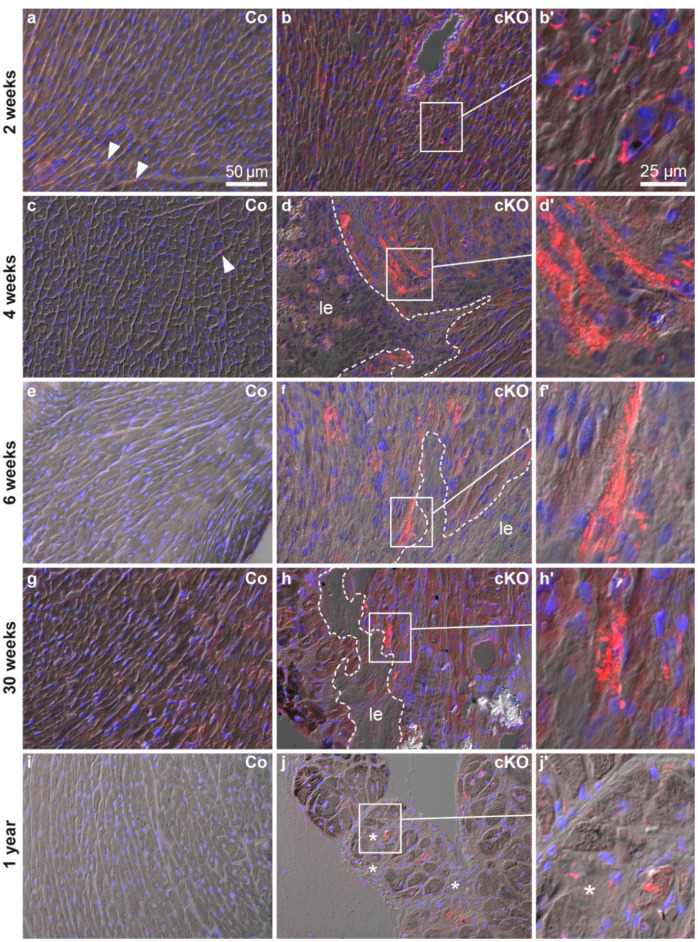
The ubiquitin binding protein SQSTM1/p62 is expressed in a similar temporal and spatial pattern as LC3 (compare with Figure 1). All micrographs are taken from left ventricles. The images show the merged pictures of the blue channel (nuclear Hoechst 3342 stain), the red channel (immunohistochemical detection of SQSTM1/p62 protein) and differential interference contrast. Left column: *Dsg2^flox(E4−5^*^)^ control myocardium (Co, **a,c,e,g,i**); middle and right columns: *Dsg2^cKO^* myocardium (cKO). SQSTM1/p62-positive vacuoles are scarce in the myocardium of wild-type mice of all ages (arrowheads; also refer to Figure S5), whereas abundant dot-shaped SQSTM1/p62 staining occurs in the ventricular myocardium of *Dsg2^cKO^* mice in an age- and topology-dependent fashion. Cardiomyocytes with perinuclear SQSTM1/p62-positive dots are detectable in 2 week-old *Dsg2^cKO^* hearts (**b**,**b′**). Cardiomyocytes containing vast amounts of SQSTM1/p62-positive granules appear in the vicinity of maturing fibrotic scars (le; dotted lines) at the age of 4–6 weeks (**d**,**d′**,**f**,**f′**). Such strongly SQSTM1/p62-positive cardiomyocytes next to lesions or interstitial fibrosis (le, *, respectively) are less numerous in *Dsg2^cKO^* mice at 30 weeks and 1 year (**h**,**h′**,**j**,**j′**). Scale bars: 50 µm in (**a**) (same magnification in entire left and middle columns); 25 µm in (**b′**) (same magnification in entire right column).

SQSTM1/p62 participates in selective autophagy by guiding substrates into autophagosomes [41,42] and anti-SQSTM1/p62 stainings have been reported to coincide with anti-LC3 stainings [43,44]. SQSTM1/p62 localization was studied on cryosections of 2, 4, 6, 30 and 52 week-old *Dsg2^cKO^* and control mice. Cryomaterial of 12 week-old mice was not available. We detected SQSTM1/p62-positive granules in cardiomyocytes and non-myocardial cells of normal-appearing myocardium in a 2 week-old *Dsg2^cKO^* mouse (Figure 2b,b′). In control hearts, SQSTM1/p62 granules were rarely seen (Figure 2a). Cardiomyocytes with abundant SQSTM1/p62-positive aggregates were noted at 4 and 6 weeks in *Dsg2^cKO^* hearts. These cells were located next to developing replacement fibrosis (Figure 2d,d′,f,f′). Control hearts only occasionally presented SQSTM1/p62-positive cells (Figure 2c,e and Figure S5). Of note, SQSTM1/p62-positive cells were detected during the chronic disease phase in *Dsg2^cKO^* hearts at 30 weeks and one year (Figure 2h,h′,j,j′), but not in control hearts (Figure 2g,i). However, the frequency was lower than during the acute phase (Figure 2d,f).

**Figure 3:**
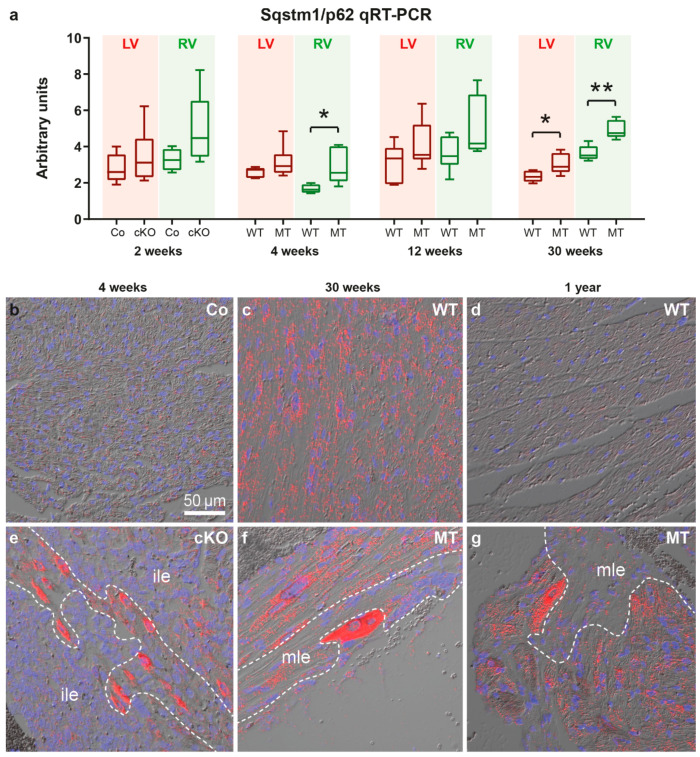
Sqstm1/p62 mRNA expression is elevated at disease onset and during chronic disease progression in *Dsg2*-mutant hearts. (**a**) The Tukey’s whisker plots depict p62 mRNA expression determined by qRT-PCR in the right ventricle (RV) and left ventricle (LV) of *Dsg2^cKO^* (cKO) and homozygous *Dsg2^flox(E4−5)^* control (Co) mice aged 2 weeks (prior to macroscopic lesion formation) as well as *Dsg2^MT^* (MT) and wild-type (WT) mice at the indicated time points of the acute and chronic disease phase. The non-parametric Mann-Whitney test was applied (* *p* ≤ 0.05; ** *p* ≤ 0.01). Further details of the statistical evaluation are provided Supplemental Tables S1 and S2. (**b**–**g**) show representative results of Sqstm1/p62 mRNA in situ hybridization. (**b**–**e**,**g**) show left ventricles, (**g**) depicts a right ventricle. Red dots indicate the presence of Sqstm1/p62 mRNA. The red channel is merged with the corresponding differential interference contrast image and nuclear Hoechst 33,342 detection (blue). Note the increased Sqstm1/p62 mRNA expression in cardiomyocytes located next to immature myocardial lesions (ile, (**e**)) and matured fibrotic lesions (mle, (**f**,**g**)), which is in agreement with the SQSTM1/p62 immunodetection in Figure 2. Positive and negative control experiments are shown in Figures S6–S8. Scale bar: 50 µm in b (same magnification in all images).

To gain insight into the mechanisms of SQSTM1/p62 protein accumulation, cardiac Sqstm1/p62 mRNA expression was assessed by qRT-PCR and in situ hybridization (Figure 3; controls in Figures S6–S8). Sqstm1/p62 mRNA was significantly elevated in right ventricles of 4 week-old and in both ventricles of 30 week-old *Dsg2*-mutant mice. Furthermore, there was a trend towards a higher Sqstm1/p62 mRNA expression in right ventricles of 2 and 12 week-old mutants (Mann Whitney tests: *p* = 0.111 and *p* = 0.0734, respectively). Sqstm1/p62 mRNA accumulated in individual cardiomyocytes that were localized next to or within forming scar tissue and adjacent to established replacement scars and strands of interstitial fibrosis (Figure 3e,f) coinciding with SQSTM1/p62 protein distribution (Figure 2). Remote myocardium, which is the healthy-appearing mutant myocardium far away from necrotic/inflamed areas, fibrotic scars or interstitial fibrosis, and the myocardium of healthy controls presented only low-level and scattered Sqstm1/p62 mRNA signals indicating a basal expression level in healthy cardiac cells. Of note, our experimental setup allowed semi-quantitative assessment of the in situ hybridization signal intensity only within an age group but not between the studied age groups. Since tissue samples of the three age groups were processed on three different days the visible differences in signal intensity, especially in the wild type (Figure 3b–d), are most likely a consequence of differences in tissue digestion and signal amplification. Furthermore, age-dependent differences in tissue structure resulted in different detectability of the dotted hybridization signal in the overlays with the differential interference images.

### 3.3. Ultrastructural Alterations Occur in Dsg2-Mutant Mice during Disease Onset and Chronic Disease Progression

**Figure 4:**
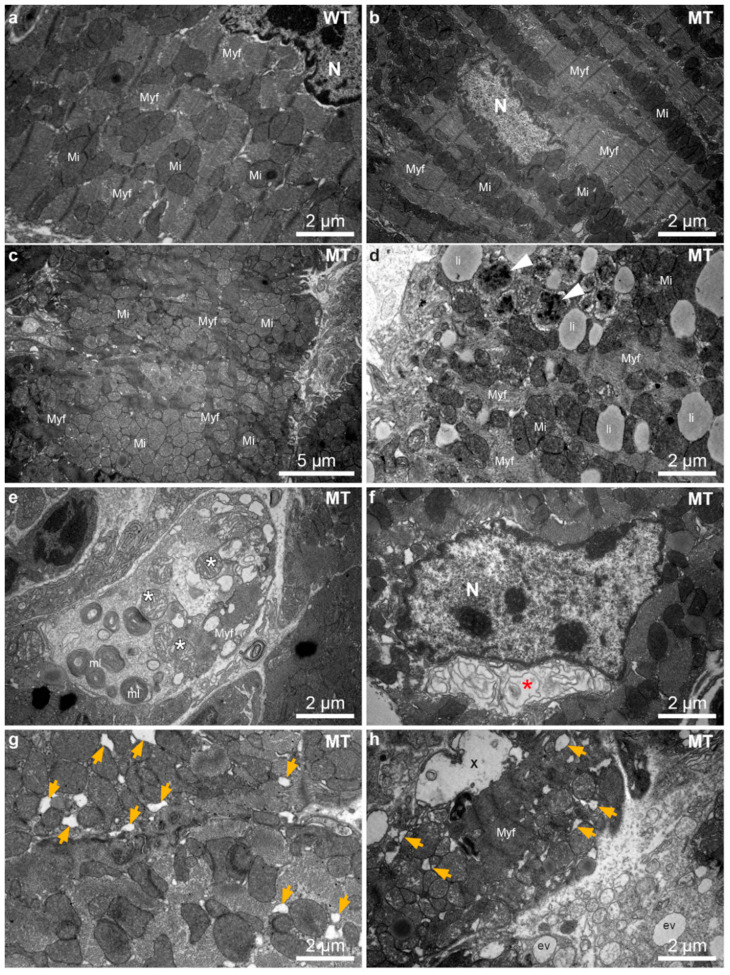
*Dsg2^MT^* (MT) hearts contain numerous cardiomyocytes with architectural disorders, multilamellar bodies and autophagosomes at structural disease onset (2–3 weeks). (**a**–**e**,**g**,**h**) show micrographs from the left and (**f**) a micrograph from the right ventricle. (**a**) shows an electron micrograph of a typical wild-type (WT) cardiomyocyte. Mi, mitochondrium; Myf, myofibril; N, nucleus. (**b**) shows the ultrastructure of a normal-appearing cardiomyocyte of a *Dsg2^MT^* (MT) mouse. A considerable number of cardiomyocytes, however, present abnormalities such as mitochondrial clustering, myofibrillar misalignment and degeneration (**c**,**d**). Autophagic vacuoles (white arrowheads, (**d**)) and multilamellar bodies (mL, (**e**)) are also detectable in the *Dsg2^MT^* myocardium. Lipid droplets (li) are found in mutant and wild-type cardiomyocytes. An extensive myofibril-free region of a cardiomyocyte and mitochondria with rarefied cristae (*) of a *Dsg2^MT^* hearts are shown (**e**). An enlarged, membrane-containing perinuclear space (*) is shown in (**f**). It is indicative of autosis. Dilated ER/SR cisternae ae observed in cardiomyocytes of *Dsg2^MT^* mice (orange arrows in (**g**,**h**)). Furthermore, cardiomyocytes containing empty ballooned structures (X) and areas with amorphous cytoplasm and empty vacuoles (ev) are detectable (**h**).

Structural indicators of autophagy such as multilamellar bodies, autophagosomes and multivesicular bodies were previously described in *Dsg2^MT^* hearts [24]. It is also known that endoplasmic reticulum (ER) stress is one inducer of autophagy and manifests structurally as enlargement of ER and sarcoplasmic reticulum (SR) cisternae [45,46,47]. To find out whether autophagy and ER stress play a role at disease onset, 1243 electron microscopic pictures of 8 *Dsg2^MT^* mice, 3 *Dsg2^WT^* mice and one *Dsg2^mt/wt^* mouse aged 2 weeks and one *Dsg2^MT^* and one *Dsg2^mt/wt^* control mouse aged 3 weeks were analyzed (Figure 4). Hearts of *Dsg2^MT^* mice presented numerous cardiomyocytes with architectural disorders such as mitochondrial clustering and myofibrillar misalignment (Figure 4c,d). In addition, degenerated myofibrils were found (Figure 4c–e). Signs of autophagy, i.e., autophagic vacuoles and multilamellar bodies, were more common in mutant (Figure 4d,e) than in wild-type cardiomyocytes (Figure 4a). Furthermore, extensive myofibril-free regions were only found in *Dsg2^MT^* cardiomyocytes (Figure 4e). In some instances, mutant cardiomyocytes presented an enlarged, membrane-filled perinuclear space (Figure 4f) which is similar to regions observed in autosis as recently reported in the context of ischemic/reperfusion injury of the heart [48]. Cardiomyocytes with empty ballooned structures, amorphous cytoplasm and empty vacuoles are also indicative of autosis (Figure 4h; [48]). Finally, dilated ER/SR cisternae were observed much more often in cardiomyocytes of *Dsg2^MT^* mice at structural disease onset (Figure 4g,h) than in control hearts (Figure 4a).

**Figure 5:**
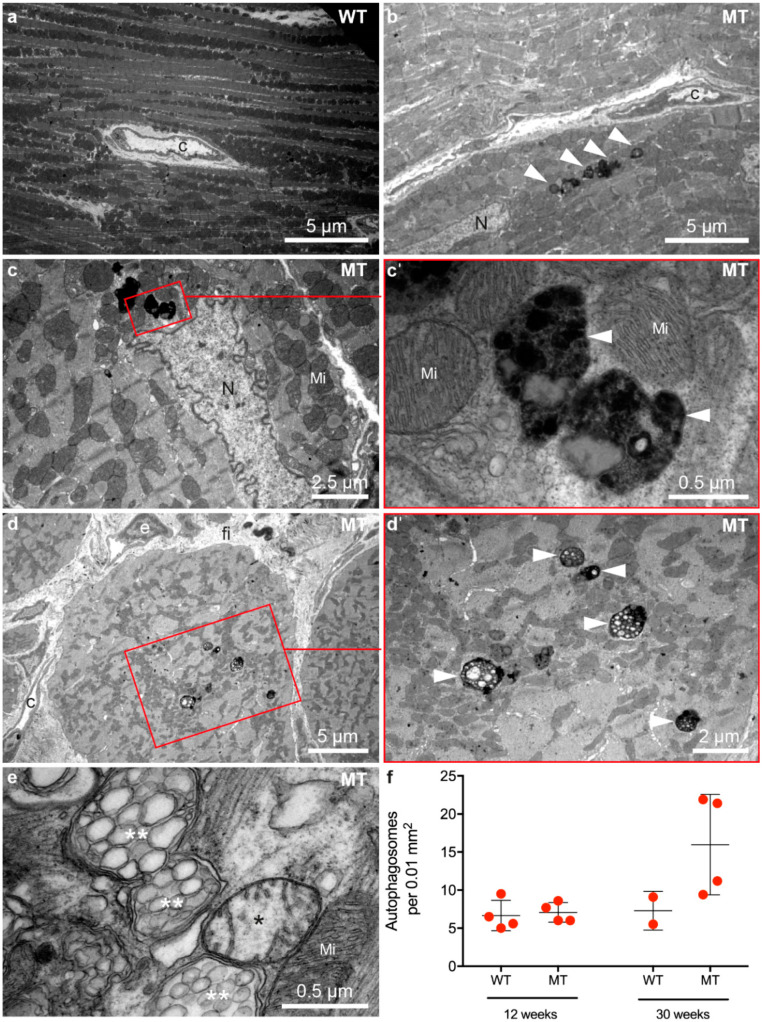
Cardiomyocytes contain an increased number of autophagosomes and autolysosomes during the chronic phase of AC. Micrographs in (**a**,**b**,**d**,**e**) show right ventricles and (**c**) shows the left ventricle. Transmission electron microscopy reveals that cardiomyocytes of 30 week-old *Dsg2^MT^* mice (MT) contain more autophagosomes and autolysosomes (white arrowheads; (**b**–**e**,**c′**,**d′**) are magnifications of the marked area an (**c**,**d**), respectively) than wild-type controls ((**a**); WT). The autophagosomes contain electron-dense cytoplasmic components and degenerated cellular organelles. (**e**) Mitochondria with rarefied cristae (*) and swollen mitochondria with vesicular cristae (**) indicate mitochondrial damage during chronic disease progression. C, capillary; fi, fibrosis; Mi, normal mitochondrion; N, nucleus. Quantification of autolysosomes at the age of 12 and 30 weeks is shown in (**f**). Due to the limited number of examined animals no statistical analysis was carried out. Representative electron microscopic images of 12 week-old mice are depicted in Supplemental Figure S9.

Autophagic vacuoles, encompassing autophagosomes and autolysosomes were also present in hearts of *Dsg2^MT^* mice at the end of the acute phase and during chronic disease progression. To quantify their number, 2218 electron microscopic images from 8 *Dsg2^MT^* (N = 8) and 1234 images from 6 *Dsg2* wild-type mice (N = 6) aged 12 and 30 weeks were systematically analyzed (examples in Figure 5 and Figure S9). While the number of autophagic vacuoles did not differ between mutant and control myocardium at 12 weeks (Figure 5f and Figure S9), a higher number of autophagic vacuoles was detected in *Dsg2^MT^* hearts at the age of 30 weeks (Figure 5a–f). The majority of these autophagic vacuoles contained electron-dense material most probably resembling organelles at different stages of degeneration classifying the vacuoles more precisely as autolysosomes [39]. Next to autolysosomes, mitochondria could be found at different stages of degeneration in the myocardium of *Dsg2^MT^* mice (Figure 5d). Of note, the detected ultrastructural signs of autophagy are consistent with immunostainings of LC3 (Figure 1g–j′) and Sqstm1/p62 (Figure 2g–j′).

**Figure 6:**
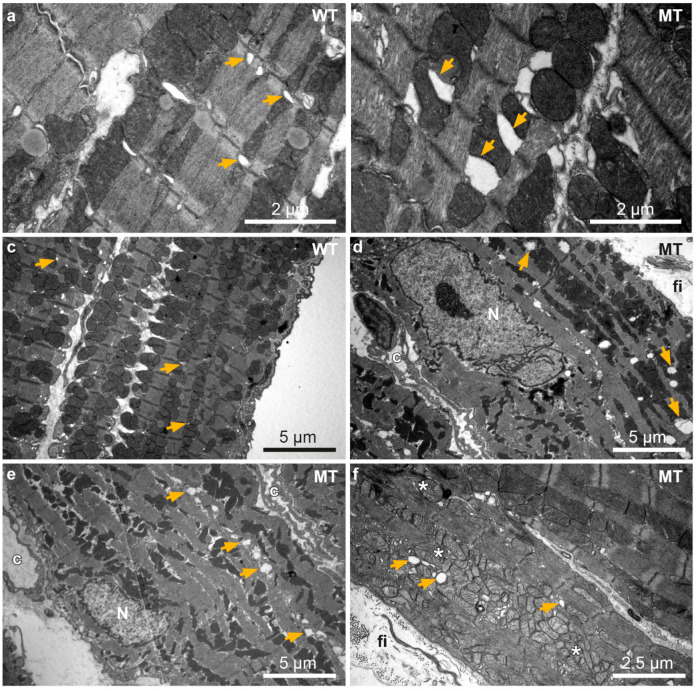
Ultrastructural analysis reveals sarcoplasmic reticulum (SR) dilation in *Dsg2^MT^* hearts. Transmission electron microscopy images of 12 and 30 week-old wild-type (WT; (**a**,**c**)) and *Dsg2^MT^* mice (MT; (**b**,**d**–**f**)) are shown. (**a**,**b**) show left and (**c**–**f**) right ventricles. Cardiomyocytes with enlarged ER/SR lumina are occasionally detected in *Dsg2^MT^* mice at 12 weeks ((**b**); orange arrow). At the age of 30 weeks more cardiomyocytes of *Dsg2^MT^* mice present dilated ER/SR (orange arrows) and mitochondria with rarefied cristae (*; (**d**–**f**)). Note that cardiomyocytes with dilated ER/SR and/or mitochondria are located adjacent to strands of interstitial fibrosis (fi). Capillaries are marked with “c”.

Ultrastructural analyses by transmission electron microscopy furthermore revealed dilation of ER/SR cisternae only in a few cardiomyocytes of 12 week-old *Dsg2^MT^* mice (Figure 6b). An increasing number of cardiomyocytes with enlarged ER/SR cisternae was detected during progression of the disease as exemplified in Figure 6d–f for 30 week-old *Dsg2^MT^* hearts. ER/SR dilation occurred preferentially in cardiomyocytes that presented additional structural abnormalities such as accumulations of autolysosomes (Figure 6d), aberrantly shaped mitochondria (Figure 6e) and mitochondria with rarefied cristae (Figure 6f).

### 3.4. Markers of the Unfolded Protein Response Are Increased at Disease Onset

**Figure 7:**
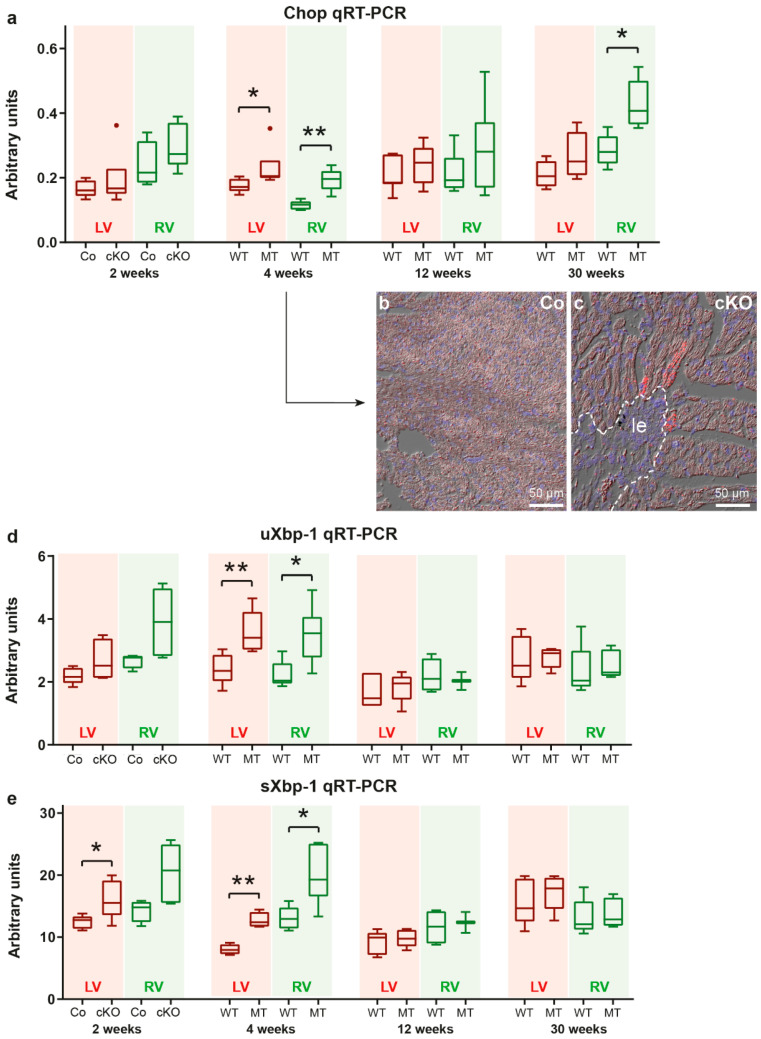
Molecular markers of ER stress and the unfolded protein response (UPR) are upregulated in a defined temporal and spatial manner in *Dsg2^cKO^* (cKO) and *Dsg2^MT^* (MT) hearts. (**a**) Tukey’s whisker plots of Chop mRNA expression in the right and left ventricles (RV and LV, respectively) of *Dsg2^cKO^* (cKO) and *Dsg2^flox(E4−5)^* control mice (Co) aged 2 weeks and *Dsg2^MT^* (MT) and wild-type (WT) mice at the indicated time points using qRT-PCR. (**b**,**c**) Representative in situ hybridization of Chop mRNA in cardiac sections of 4 week-old *Dsg2^cKO^* mice and *Dsg2^flox(E4−5)^* control mice (Co). The micrographs consist of three different channels: red, Chop mRNA; blue, nuclear Hoechst 3342 staining and differential interference contrast. Left ventricles are shown. Chop mRNA is highly expressed in some cardiomyocytes adjacent to fibrotic scars (le, demarcated by a dotted line). The Chop in situ hybridization micrographs of other ages are depicted in Supplemental Figure S10. (**d**,**e**) Tukey’s whisker plots of sXbp1 and uXbp1 mRNA expression. Elevated sXbp1 mRNA levels are present in left ventricles of 2 week-old *Dsg2^cKO^* mice as well as in left and right ventricles of 4 week-old *Dsg2^MT^* mice indicating activation of the unfolded protein response. The non-parametric Mann-Whitney test was applied to compare cKO/MT and Co/WT expression at each single time point in (**a**,**d**,**e**) (* *p* ≤ 0.05; ** *p* ≤ 0.01).

The unfolded protein response (UPR) is activated during ER stress and induces signaling pathways that transmit the stress signal to the cytoplasm to restore protein homeostasis. One important molecule in this process is the transcription factor CHOP (encoded by the Ddit3 gene), a direct regulator of Map1lc3a (LC3) and Sqstm1/p62 gene expression [49,50,51]. We found significantly elevated Chop mRNA expression in the right and left ventricles of 4 week-old *Dsg2^MT^* mice (Figure 7a). In situ hybridization furthermore revealed that Chop mRNA expression is highest in some cardiomyocytes that are in close proximity to areas of replacement fibrosis (Figure 7 b,c and Figure S10). Chop mRNA expression declined to wild-type levels by week 12 in both ventricles of *Dsg2^MT^* mice but rose again in right ventricles during chronic disease progression (30 weeks in Figure 7a).

Since Chop overexpression may induce apoptosis [52], we analyzed cleaved caspase 3 (CC3) protein expression by immunohistochemistry in the hearts of 18 day-old *Dsg2^cKO^* mice and hearts of 4 and 30 week-old *Dsg2^MT^* mice (Figure S11). The immune cell infiltrates surrounding necrotic cardiomyocytes detected in the 18 day-old *Dsg2^cKO^* hearts contained CC3-positive cells (Figure S11a,d,g), but no CC3-positive cardiomyocytes were found. In the hearts of 4 week-old (Figure S11b,e,h) and 30 week-old *Dsg2^MT^* mice (Figure S11c,f,i) only very few single CC3-positive non-cardiomyocytes (1–2 cells per section) were detected.

Another branch of UPR signaling involves the splicing of the X-box binding protein mRNA (uXbp1 mRNA) into the spliced Xbp1 (sXbp1) mRNA. Detection of sXbp1 mRNA is therefore a well-established and reliable marker of ER stress induction [46,53]. We found that sXBP1 mRNA expression is already elevated in left and right ventricles of 2 week-old *Dsg2^cKO^* mice, i.e., prior to structural disease onset (Figure 7e). In addition, uXbp1 mRNA shows a trend towards elevated expression levels in the right ventricle (Mann-Whitney test: *p* = 0.0635). At the age of 4 weeks, elevated uXbp1 and sXbp1 mRNA expression is detected in both heart chambers of *Dsg2^MT^* mice (Figure 7d,e). Later on, uXbp1 and sXbp1 mRNA levels were not different from those in the wild-type (12 weeks and 30 weeks in Figure 7d,e).

### 3.5. mRNAs Coding for Calcium Handling Proteins Are Dysregulated during the Chronic Disease Phase

**Figure 8:**
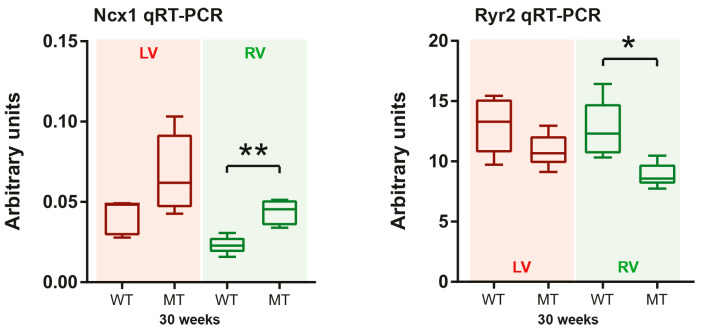
Ncx1 and Ryr2 mRNA expression is predominantly impaired in right ventricles during chronic AC progression. Tukey’s whisker plots depict the mRNA expression in right and left ventricles (RV and LV, respectively) of *Dsg2^MT^* (MT) and wild-type mice (WT) as determined by qRT-PCR. The non-parametric Mann-Whitney test was applied (* *p* ≤ 0.05; ** *p* ≤ 0.01). The mRNA expression of all other assessed time points and detailed statistical analyses are available in Supplemental Tables S1 and S2.

Changes of intracellular calcium homeostasis are potential activators of autophagy [21] and calcium-dependent signaling can be induced by ER stress [54]. We already reported previously that mRNAs coding for phospholamban, a regulator of calcium uptake into the SR, and the SR calcium pump SERCA2 (Atp2a2a mRNA) are reduced in right and left ventricles of 4 week-old *Dsg2^MT^* mice [26]. Afterwards, Atpa2a (Serca2a) mRNA expression decreases in right ventricles but recovers in left ventricles during AC progression [26]. Here, we examined the ventricle-specific mRNA expression of the ryanodine receptor 2 (Ryr2), which is a SR calcium channel, and the sodium-calcium exchanger 1 (Ncx1) in *Dsg2* mutant mice. The encoded proteins are involved in the regulation of ER/SR and cytoplasmic calcium homeostasis. Ryr2 and Ncx1 mRNA expression did not differ between *Dsg2^cKO^*, *Dsg2^MT^* and the respective control mice at 2, 4 and 12 weeks (Tables S1 and S2). However, Ncx1 mRNA expression was increased and Ryr2 mRNA expression was decreased in the hearts of 30 week-old *Dsg2^MT^* mice (Figure 8). The changes were statistically significant only in right ventricles. Together, the changes may lead to imbalances of cytoplasmic and ER/SR calcium homeostasis [55,56,57].

## 4. Discussion

**Figure 9:**
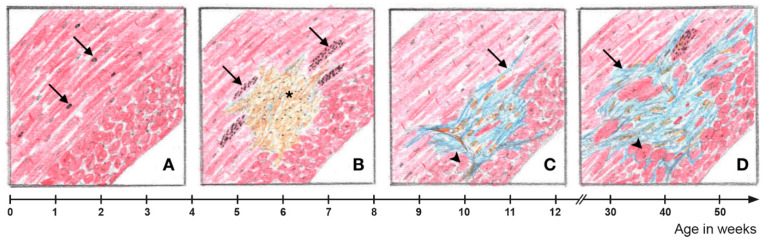
A spatial relationship exists between myocardial fibrosis and cardiomyocytes with increased autophagic activity in *Dsg2*-mutant hearts. (**A**) Prior to structural disease onset, some *Dsg2*-mutant cardiomyocytes in the circular myocardium, which experience high mechanical stress, show signs of increased autophagic activity as evidenced by isolated autophagic vacuoles (arrows). (**B**) Focal cardiomyocyte necrosis occurs at the onset of the acute phase of AC progression (*). Cardiomyocytes with markedly increased activity of autophagy and ER stress can be found in the vicinity of cell-rich lesions (arrows). (**C**) Connective tissue remodeling of the myocardial lesions is completed at the end of the acute phase. In the area of mature replacement fibrosis, incipient hypertrophy of adjacent cardiomyocytes can be detected (arrowhead). In addition, interstitial fibrosis becomes apparent (arrow). (**D**) Interstitial fibrosis expands during the chronic AC phase. There is also distinct hypertrophy of cardiomyocytes in the vicinity of fibrotic myocardial lesions. Signs of ER stress and increased autophagic activity are again detectable in perilesional cardiomyocytes.

Autophagy is an adaptive physiological reaction of cardiomyocytes to cellular stress [58,59,60]. It is important for cardiac developmental and maturation processes [61]. Dysregulation of autophagy, caused by pathological stress, plays a pivotal role in the manifestation of various cardiac diseases [21,22,23,41,62,63,64,65]. In this study, we examined whether autophagy is also impaired in AC by focusing on local changes that are associated with focal lesions that appear during disease inception and progression in *Dsg2^MT^* and *Dsg2^cKO^* mice. We found an accumulation of LC3 and Sqstm1/p62 in circumscribed sites during distinct stages of AC manifestation that coincided with ER/SR stress marker expression and ultrastructural ER/SR dilation (schematic summary in Figure 9). Most remarkably, we detected signs of increased autophagy prior to structural disease onset in the normal-appearing mutant myocardium. We furthermore found that cardiomyocytes, which are completely filled with LC3- and SQSTM1/p62-positive granules, line the forming scars during the acute phase. Finally, we identified cardiomyocytes with signs of increased LC3 and Sqstm1/p62 expression during the chronic disease phase next to replacement and interstitial fibrosis but not in normal-appearing myocardium, which was remote from lesions. In the following sections we will discuss these disease phase-specific findings in more detail.

### 4.1. Enhanced Autophagy and Endoplasmic Reticulum Stress May Be Induced by Altered Force Distribution

Increased LC3 and SQSTM1/p62 expression together with elevated sXbp1 mRNA expression occur in 2 week-old Dsg2-mutant myocardium at a time, when slightly enhanced autophagy is also detectable in control wild-type myocardium. The latter observation signifies myocardial maturation and remodeling, which takes place during this early postnatal developmental phase [66,67]. The pronounced increase of autophagic markers in histologically normal-appearing Dsg2-mutant hearts may therefore indicate that the changes in Dsg2 expression compromise the physiologically occurring structural and biochemical maturation processes, which involve heart growth, remodeling of the contractile apparatus and T tubules, ER/SR metabolism, membrane organization and intercalated disc structure [66,67,68,69,70,71]. The reorganizing myocardium may be particularly sensitive and responsive to mechanical perturbation during these postnatal maturation processes [72].

Compromising effects of *Dsg2* mutation on cell mechanics are most likely mediated through altered desmosomal cell-cell adhesion at the intercalated disc and changes in the three-dimensional organization of the desmosome-anchored desmin intermediate filament network. The latter forms a complex scaffold for organelles and myofibrils [73,74,75]. The recently observed considerably altered desmin organization in *Dsg2* mutants during embryonic cardiogenesis with detrimental consequences on cardiac differentiation and function underscores the importance of an intact desmin network for cellular integrity [76]. It is therefore safe to assume that disturbances of the three dimensional desmin network in the *Dsg2*-mutant hearts also interfere with proper postnatal myocardial maturation [74]. In support, our electron microscopic studies of two week-old *Dsg2^MT^* mice revealed myofibrillar misalignment, myofibril-depleted area, which were sometimes filled with multilamellar bodies, and mitochondrial clustering. The perturbed cardiomyocyte maturation is most likely the reason for the increased autophagy. The dilated ER/SR cisternae furthermore indicate that these dysfunctions are accompanied by ER stress.

Cardiomyocyte cohesion is severely impaired during late disease stages in *Dsg2*-mutant mice [33]. The compromise in desmosomal cell-cell adhesion interferes with effective longitudinal force transmission through the linked desmin network and therefore reduces the absorption of diastolic strain forces. This may, in turn, lead to increased strain on myofibrillar proteins and, hence, to an increase of mechanically damaged proteins [77]. This may overload the repair capacity of the maturing *Dsg2*-mutant cardiomyocytes and induce cardiomyocyte death. This idea is supported by recently published experimental data showing that diastolic lengthening is crucial to induce the AC phenotype [78]. However, the mechanisms leading to subsequent necrotic cardiomyocyte death remain to be worked out.

### 4.2. Endoplasmic Reticulum Stress and Increased Autophagy in Cardiomyocytes Coincide with Fibrotic Lesion Progression

The observed massive increase of LC3 and SQSTM1/p62 protein expression in single cardiomyocytes in the vicinity of forming scar tissue can be taken as evidence for either a local pathological increase of autophagy or a blockade of autophagy with toxic accumulation of autophagic vacuoles. We propose that perilesional cardiomyocytes experience disparate mechanical loads since they are trapped between the non-contractile maturing scar tissue and the contractile myocardium which is expected to exert mechanical stress on cardiomyocytes leading to the activation of mechanical stress sensors [79,80,81]. Furthermore, the paracrine micromilieu is impaired in the microenvironment of cardiomyocytes lining the forming fibrous scar tissue due to the presence of inflammatory immune cells [28,36]. Inflammation triggers oxidative stress, which is a known activator of autophagy [82]. We suggest that lesion-lining cardiomyocytes, which are completely packed with LC3- and SQSTM1/p62-positive vacuoles, are destined for cell death. This, in turn, leads to scar expansion. Since CC3-positive cardiomyocytes are extremely rare in the *Dsg2* mutant hearts, apoptosis can be excluded as a major mode of cell death. While we and others classified the primary mode of cell death in *Dsg2*-mutant mice as necrosis based on the morphotype of dying cardiomyocytes in hematoxylin/eosin-stained tissue sections [28,37], the mechanism of cell death in these lesion-lining cardiomyocytes may also involve autophagy-dependent cell death or autosis [83]. Our electron microscopical analyses of 2–3 week old *Dsg2*-mutant mice support this possibility. Interestingly, autosis also occurs in cardiomyocytes lining areas of ischemic/reperfusion injury [48].

The observed elevated Chop mRNA expression in cardiomyocytes next to lesions indicates local ER stress, which is further supported by the overall increase of uXbp1 and sXbp1 mRNA expression during scar formation in the early acute phase [46,50,84]. The ER/SR dilation in cardiomyocytes of *Dsg2*-mutant mice is another indication of ER/SR stress that has been described also in another *Dsg2*-mutant based AC mouse model [25]. It is also supported by the recent observation of reduced Atpa2a (Serca2a) and phospholamban mRNAs, whose encoded proteins are involved in SR-mediated calcium handling [26]. Further investigations are needed to mechanistically link desmosomal mutations with ER stress and the induction of autophagy in murine AC pathogenesis.

### 4.3. Local ER Stress and Autophagy Are Induced in Cardiomyocytes Adjacent to Mature Fibrotic Scars and Interstitial Fibrosis

Strikingly, we observed that ER/SR stress markers normalize in young adult mice with fully matured hearts and fully developed scar tissue (10–12 weeks) but increase again in older animals, especially in the right ventricular wall. We take this as evidence for deteriorating ER/SR function during chronic disease progression, which goes along with reduced ryanodine receptor 2 expression and was also described in conjunction with plakophilin 2 mutation [85,86]. In addition, reduced Serca2a expression was observed in right ventricles of *Dsg2^MT^* mice during chronic AC progression [26], in the failing right ventricles of AC patients [26] and other cardiomyopathies [87]. It goes along with an inability to normalize cytosolic calcium levels during diastole [88]. The observed enhanced Ncx1 mRNA expression is most likely part of a compensatory reaction to regulate cytoplasmic calcium levels (for other cardiomyopathies see [87]).

The altered expression of calcium handling proteins is in accordance with changes in intracellular Ca^2+^ dynamics, which are a known hallmark of AC. They were studied and visualized in murine cardiomyocytes [89,90] as well as in human iPSC-derived cardiomyocytes [91,92,93,94]. Alterations included arrhythmogenic events such as early after depolarizations (EAD) and delayed after depolarizations (DAD) [89,93], reduced time to peak of intracellular Ca^2+^ [94] and a reduced conduction velocity in affected myocardium [92]. In the case of murine cardiomyocytes, an increased frequency of spontaneous Ca^2+^ release events was observed [89,90]. Some of these changes might be responsible for the susceptibility to arrhythmias characterizing the clinical phenotype of AC [86,95,96,97].

Similar to the acute disease phase, cardiomyocytes with the highest Sqstm1/p62 and LC3 protein expression were located adjacent to fibrotic altered myocardium during chronic disease progression, albeit at much lower levels. Interestingly, myocardium in proximity to fibrotic scar tissue is known to be a hotspot for the induction of arrhythmia [77,98]. We recently reported that especially cardiomyocytes next to fibrosis undergo pathological hypertrophy as evidenced by an increased diameter, ectopic expression of α skeletal muscle actin (ACTA1) and activation of SRF signaling in *Dsg2* mutants [26,99]. Due to their increased diameter, mutant cardiomyocytes also suffer from functional hypoxia and/or oxidative stress [82]. Thus, cardiomyocytes adjacent to fibrotic scars are under multiple stresses, which go along with increased ER/SR stress and the induction of autophagy, a phenomenon also reported in desmin-related cardiomyopathies [42]. Exhaustion of the autophagic capacity of cardiomyocytes next to lesions may then result in cell death. This, in turn, leads to an increase in interstitial fibrosis and chamber dilation driving the heart into failure as is also the case in other cardiomyopathies [82,98].

## 5. Conclusions

Altogether, we conclude that ER/SR stress and autophagy are pathological traits in *Dsg2*-related cardiomyopathy. The alterations are topologically restricted to specific cardiac tissue compartments and can be separated into different phases, each with a unique signature dependent on the developmental status of the heart and pathogenic progression. However, detailed analyses are still needed to work out mechanistic links between desmosomal mutations, ER stress induction, the type of unfolded protein response, autophagic flux rates and the exact type of cell death during the different AC disease phases.

## Figures and Tables

**Table 1:** List of qRT-PCR primer pairs and universal probe library (UPL) probes.

Gene	NCBI ID	Sequence Forward (5′–3′)	Sequence Reverse (5′–3′)	UPL
Chop	NM_007837.3	GCGACAGAGCCAGAATAACA	GATGCACTTCCTTCTGGAACA	#91
Sqstm1/p62	NM_011018.3	AGACCC CTCACAGGAAGGAC	CATCTGGGAGAGGGACTCAA	#41
uXbp1	NM_013842.3	TGACGAGGTTCCAGAGGTG	TGCAGAGGTGCACATAGTCTG	#49
sXbp1	NM_001271730.1	AGCAAGTGGTGGATTTGGAA	CCGTGAGTTTTCTCCCGTAA	#78
Hmbs (reference gene)	NM_013551.2	AAGTTCCCCCACCTGGAA	GACGATGGCACTGAATTCCT	#42

## Data Availability

The data presented in this study are available on request from the corresponding authors.

## Supplementary Materials

The following items are available online at https://www.mdpi.com/article/10.3390/cells11010096/s1, 1 PDF document entitled “Supplemental data Pitsch et al.” containing 2 supplemental tables: Table S1. Left ventricular mRNA expression of Sqstm1/p62, ER stress marker (Chop, uXbp1 and sXbp1) and calcium handling proteins (Ncx1, Ryr2) assessed by qRT-PCR; Table S2. Right ventricular mRNA expression of Sqstm1/p62, ER stress marker (Chop, uXbp1and sXbp1) and calcium handling proteins (Ncx1, Ryr2) assessed by qRT-PCR; 11 Supplemental Figures: Figure S1. The time course of disease pathogenesis is very similar in *Dsg2^MT^* and *Dsg2^cKO^* mice; Figure S2. *Dsg2^cKO^* and *Dsg2^MT^* mice show similar histopathologic patterns; Figure S3. Cardiomyocytes are replaced by connective tissue in *Dsg2^cKO^* and *Dsg2^MT^* mice; Figure S4. LC3 protein expression is localized in cardiomyocytes and in the cell infiltrate surrounding necrotic cardiomyocytes of *Dsg2^cKO^* mice during structural disease onset; Figure S5. SQSTM1/p62 immunohistochemistry in healthy control animal; Figure S6. In situ hybridization controls show mRNA preservation and detection specificity in 4 week-old hearts; Figure S7. In situ hybridization controls show mRNA preservation and detection specificity in 30 week-old hearts; Figure S8. In situ hybridization controls show mRNA preservation and detection specificity in 1 year-old hearts; Figure S9. The number of autophagic vacuoles is not increased in 12 week-old *Dsg2^MT^* mice; Figure S10. Chop mRNA expression is increased in single cardiomyocytes in *Dsg2*-mutant hearts; Figure S11. Apoptotic cleaved caspase 3-positive cells are not increased in perilesional *Dsg2*-mutant myocardium.
