# Supplementary material for: Autophagy and Endoplasmic Reticulum Stress during Onset and Progression of Arrhythmogenic Cardiomyopathy
Source: Cells. 2021 Dec 29;11(1):96. doi: 10.3390/cells11010096 (PMC8750195; doi:10.3390/cells11010096)
Supplement: Supplementary file 1 [file cells-11-00096-s001.zip › cells-1481021-Supplement.pdf]

Supplements Pitsch et al., Autophagy and Endoplasmic Reticulum Stress during Onset and Progression of Arrhythmogenic Cardiomyopathy

Table S1 Left ventricular mRNA expression of Sqstm1/p62, ER stress marker (Chop, uXbp1 and sXbp1) and calcium handling proteins (Ncx1, Ryr2) assessed by qRT-PCR

	left ventricle					
	**age[&]**	***Dsg2[WT]***		***Dsg2[MT]***		***P***
**p62**	2[a]	2.814 ± 0.798	(5)	3.47 ± 1.527	(6)	0.7922
	4	2.585 ± 0.286	(5)	3.146 ± 0.873	(6)	0.1255
	12	3.204 ± 1.002	(7)	4.049 ± 1.271	(7)	0.4557
	30	2.363 ± 0.308	(5)	3.082 ± 0.567 *	(5)	0.0317
**Chop**	2 [a]	0.166 ± 0.026	(5)	0.195 ± 0.084	(6)	>0.999
	4	0.177 ± 0.021	(5)	0.23 ± 0.061 *	(6)	0.0303
	12	0.208 ± 0.051	(7)	0.235 ± 0.061	(7)	0.4557
	30	0.211 ± 0.041	(5)	0.27 ± 0.071	(5)	0.2222
**sXbp1**	2 [a]	12.43 ± 1.079	(5)	15.96 ± 3.021 *	(6)	0.0303
	4	8.03 ± 0.829	(5)	12.78 ± 1.18 **	(6)	0.0043
	12	9.118 ± 1.909	(5)	9.826 ± 1.404	(5)	0.6905
	30	52.85 ± 11.19	(5)	56.08 ± 10.55	(5)	0.5476
**uXbp1**	2 [a]	2.199 ± 0.26	(5)	2.693 ± 0.632	(6)	0.2468
	4	2.426 ± 0.49	(5)	3.597 ± 0.69 **	(6)	0.0087
	12	1.616 ± 0.474	(5)	1.83 ± 0.463	(5)	0.4207
	30	9.231 ± 2.242	(5)	9.051 ± 1.297	(5)	>0.9999
**Ryr2**	2 [a]	19.13 ± 1.276	(5)	18.95 ± 8.213	(6)	0.1255
	4	12.69 ± 5.435	(5)	8.961 ± 2.461	(6)	0.2468
	12	10.68 ± 1.999	(4)	10.59 ± 5.433	(6)	0.3524
	30	13.01 ± 2.325	(5)	10.92 ± 1.378	(5)	0.1508
**Ncx1**	2 [a]	0.065 ± 0.015	(5)	0.069 ± 0.022	(6)	0.8896
	4	0.118 ± 0.017	(5)	0.099 ± 0.033	(6)	0.1775
	11	0.044 ± 0.006	(4)	0.037 ± 0.006	(6)	0.1143
	30	*0.041 ± 0.011*	(5)	*0.068 ± 0.024*	*(5)*	0.0556

[&] age in weeks; [a] in case of 2 week-old mice *Dsg2[flox(E4-5)]* (WT) and *Dsg2[cKO]* mice (MT) were studied prior to macroscopically detectable structural disease onset (Kant et al., 2015); at all other ages *Dsg2* wildtype (WT) and constitutive *Dsg2[MT]* (MT) mice were assessed (Krusche et al., 2011); * P < 0.05, ** P < 0.01

Table S2 Right ventricular mRNA expression of Sqstm1/p62, ER stress marker (Chop, uXbp1 and sXbp1) and calcium handling proteins (Ncx1, Ryr2) assessed by qRT-PCR

	right ventricle					
	age[&]	*Dsg2^WT^*		*Dsg2^MT^*		*P*
**p62**	2[a]	*3.279 ± 0.607*	*(4)*	*4.885 ± 1.972*	*(5)*	0.1111
	4	*1.68 ± 0.237*	(5)	*2.871 ± 0.959 **	*(6)*	0.0173
	12	*3.617 ± 0.935*	*(6)*	*5.059 ± 1.581*	*(7)*	0.0734
	30	*3.64 ± 0.417*	(5)	*4.951 ± 0.51 ***	*(5)*	0.0079
**Chop**	2[a]	*0.238 ± 0.072*	*(4)*	*0.299 ± 0.071*	*(5)*	0.1905
	4	*0.115 ± 0.014*	(5)	*0.193 ± 0.034 ***	*(6)*	0.0043
	12	*0.214 ± 0.064*	*(6)*	*0.295 ± 0.128*	*(7)*	0.3660
	30	*0.285 ± 0.049*	(5)	*0.428 ± 0.075 **	*(5)*	0.0159
**sXbp1**	2[a]	*14.31 ± 1.785*	*(4)*	*20.33 ± 4.759*	*(5)*	0.0635
	4	*13.05 ± 1.847*	(5)	*19.96 ± 4.66 **	*(6)*	0.0128
	12	*11.62 ± 2.812*	*(4)*	*12.38 ± 2.383*	*(2)*	0.8000
	30	*45.95 ± 10.5*	(5)	*46.94 ± 8.195*	*(5)*	0.6905
**uXbp1**	2[a]	*2.684 ± 0.233*	*(4)*	*3.905 ± 1.081*	*(5)*	0.0635
	4	*2.228 ± 0.433*	(5)	*3.505 ± 0.886 **	*(6)*	0.0173
	12	*2.195 ± 0.536*	*(4)*	*2.035 ± 0.403*	*(2)*	0.8000
	30	*8.176 ± 2.856*	(5)	*8.648 ± 1.531*	*(5)*	0.4206
**Ryr2**	2[a]	*12.72 ± 2.387*	*(4)*	*17.38 ± 9.082*	*(5)*	0.5556
	4	*8.051 ± 2.827*	(5)	*6.803 ± 2.882*	*(6)*	0.5368
	12	*52.93 ± 28.56*	*(5)*	*57.04 ± 70.56*	*(6)*	0.1775
	30	*12.62 ± 2.384*	(5)	*8.868 ± 1.01 **	*(5)*	0.0159
**Ncx1**	2[a]	*0.039 ± 0.005*	*(4)*	*0.05 ± 0.023*	*(5)*	>0.9999
	4	*0.044 ± 0.012*	(5)	*0.062 ± 0.014*	*(6)*	0.0519
	12	*0.161 ± 0.068*	*(5)*	*0.271 ± 0.286*	*(5)*	0.6905
	30	*0.023 ± 0.005*	(5)	*0.044 ± 0.008 ***	*(5)*	0.0079

[&] age in weeks; [a] in case of 2 week-old mice *Dsg2$^{flox(E4-5)}$* (WT) and *Dsg2^{cKO}* mice (MT) were studied prior to macroscopically detectable structural disease onset (Kant et al., 2015); at all other ages *Dsg2* wildtype (WT) and constitutive *Dsg2^{MT}* (MT) mice were assessed (Krusche et al., 2011); * P < 0.05, ** P < 0.01

Figure S1. The time course of disease pathogenesis is very similar in *Dsg2*^{MT} and *Dsg2*^{cKO} mice

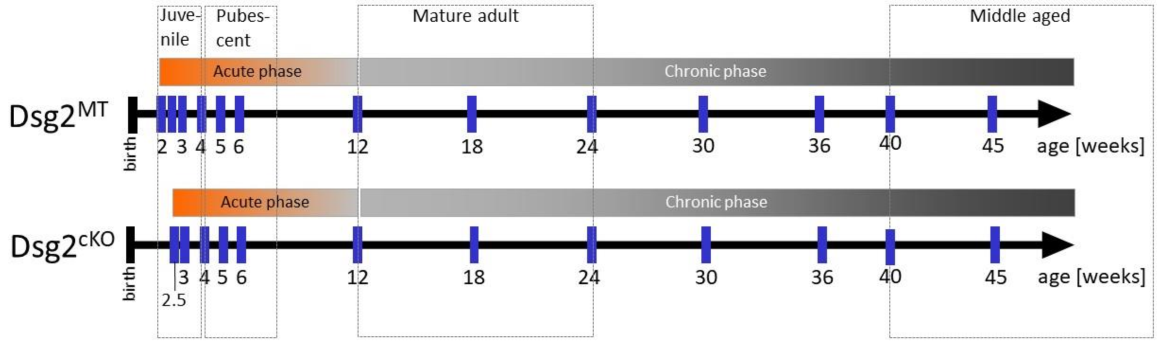

Structural disease onset, which is characterized by cardiomyocyte necrosis (orange color), occurs at the age of 2 weeks (14 ± 1-2 days) in *Dsg2*^{MT} mice and 2.5 weeks (18 ± 1-2 days) in *Dsg2*^{cKO} mice. Necrosis induces an inflammatory response similar to that observed after ischemic heart infarction. In parallel, collagen production is activated replacing lost cardiomyocytes. Scar formation may continue until the age of 8 weeks. Thereafter, the inflammatory response decreases and changes as evidenced by an altered chemokine/cytokine pattern. During the chronic disease phase (grey color) macrophages and T cells are detected in fibrous scars and in the developing interstitial fibrosis. Further information on the two mouse lines and their similarities has been published [24,26,27, 28,33].

Figure S2. ***Dsg2**^{cKO}* **and** ***Dsg2**^{MT}* **mice show similar histopathologic patterns**

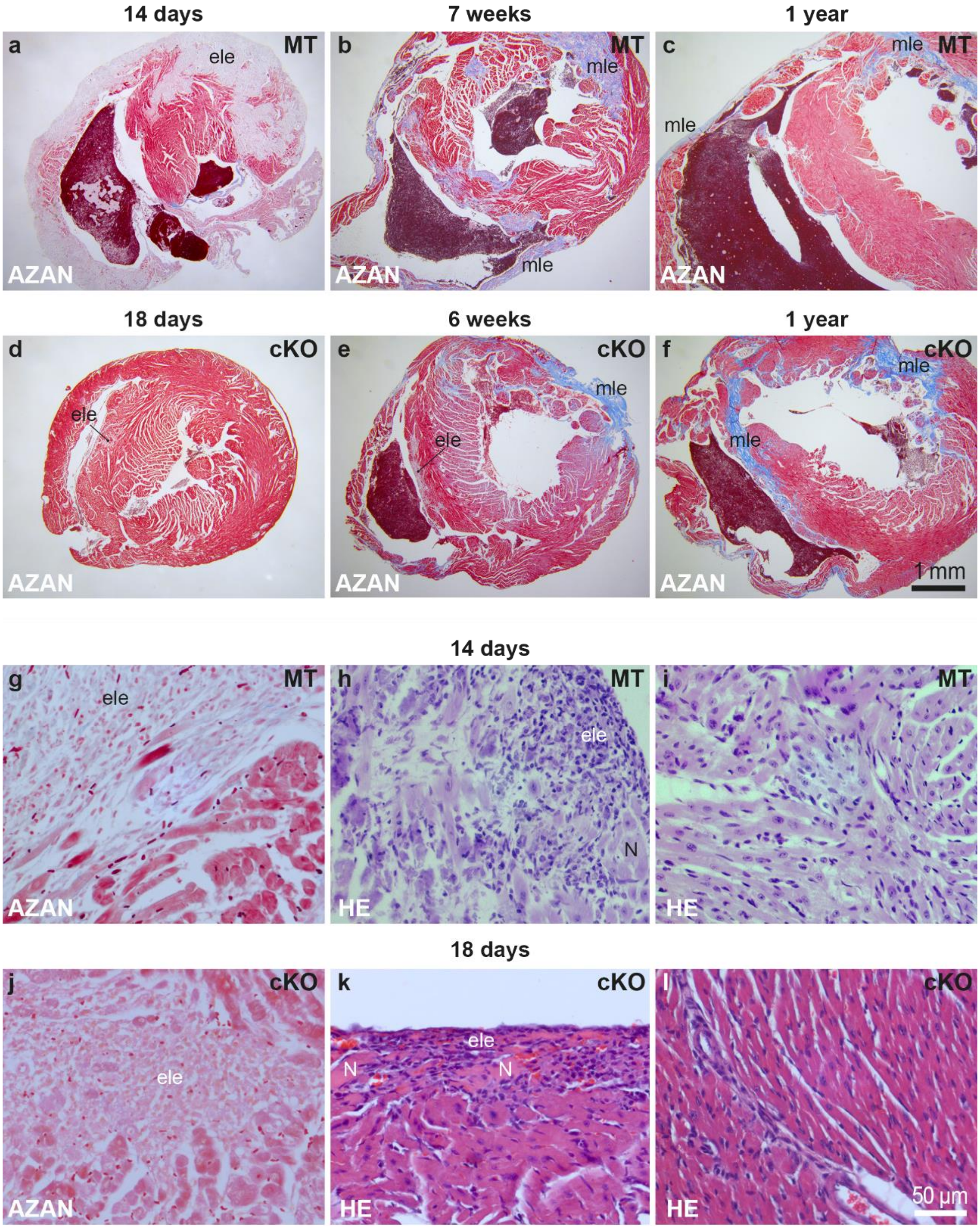

(a-f) The micrographs present survey views of cardiac sections from 14 day-old (a), 7 week-old (b) and 1 year-old (c) *Dgs2*^{MT} (MT) mice and 18 day-old (d), 6 week-old (e) and 1 year-old (f) *Dgs2*^{cKO} (cKO) mice stained with Heidenhain's AZAN trichrome stain to highlight collagenous fibers and scar tissue. (g-l) The high magnification images show the histomorphology of newly-formed lesions in *Dgs2*^{MT} (g, h) and *Dgs2*^{cKO} mice (j, k) using Heidenhain's AZAN trichrome (g, j) and hematoxylin eosin stain (h, k) and normal-appearing "remote" myocardium of both mouse strains stained with hematoxylin eosin (i, l). Early lesions (ele) contain necrotic cardiomyocytes, cellular debris and immune cell infiltrates (a, d, e, g, h, j, k). Mature lesions (mle) appear in 6 and 7 week-old *Dsg2*-mutant mice (b, e) occasionally co-existing with immature early lesions (e). Mature scars are persisting until 1 year (c, f). Size bar 1 mm in f (same magnification in a-e) and 50 µm in (l, same magnification in g-k).

Figure S3. Cardiomyocytes are replaced by connective tissue in *Dsg2^{cKO}* and *Dsg2^{MT}* mice

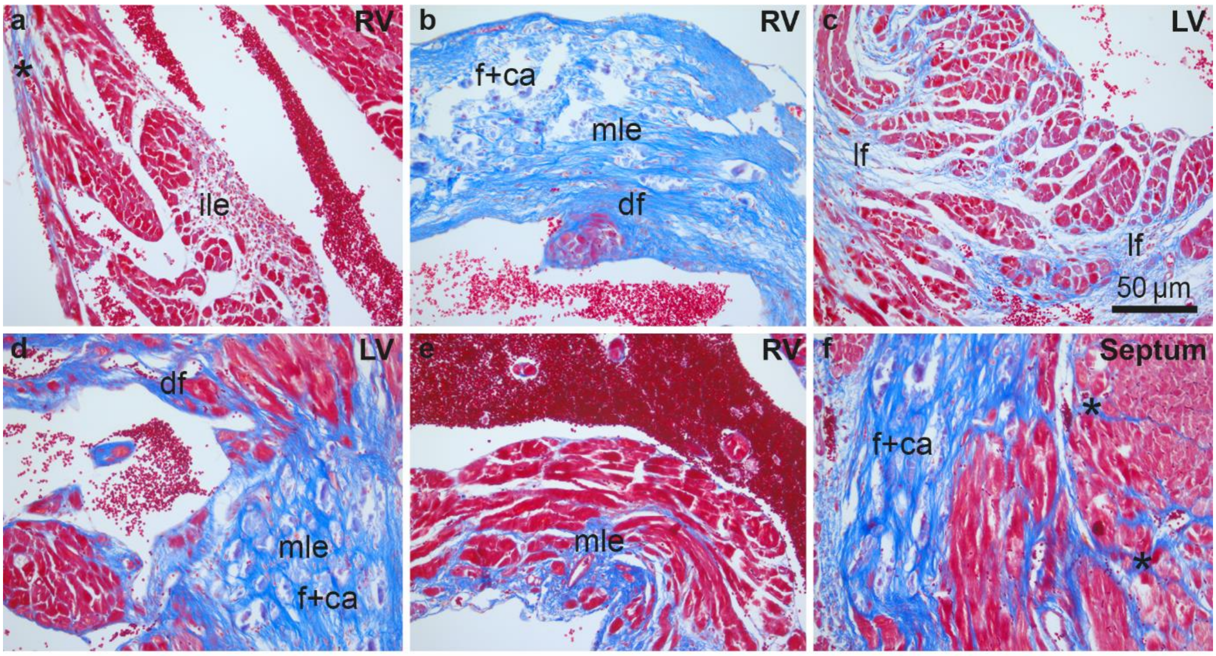

The photomicrographs show cardiac sections from 6 and (a-c) and 30 week-old (d-f) *Dgs2^{cKO}* mice stained with Heidenhain's AZAN trichrome stain to exemplify the fibrotic scars types detected in *Dsg2^{cKO}* and *Dsg2^{MT}* mice. Collagen fibers are blue. (a) depicts two lesions. While the blue purely fibrotic lesion at the left border of the right ventricle is mature (*) the lesion facing the right ventricular lumen is a newly formed immature lesion (ile) which contains many cells (red nuclei) and only few collagen fibers. The occurrence of new scars up to an age of 6-7 weeks is characteristic for the acute phase of AC which lasts from 2 - 12 weeks in *Dsg2^{MT}* and 2.5 - 12 weeks in *Dsg2^{cKO}* mice. (b) shows a mature scar that encompasses the entire right ventricular wall and consist of pure collagenous part (df) and parts with fibrosis and calcified necrotic cardiomyocytes (f+ca). (d-f) presents representative sections of 30 week-old Dsg2^{MT} and Dsg2^{cKO} mice. Only mature lesions (mle) of different types and interstitial fibrosis (*) are detectable. RV: right ventricle; LV: left ventricle; ile: immature lesion containing only few collagen fibers and many cells; mle: mature lesion with dense collagen network and few interstitial cells; df: dense fibrotic tissue; lf: loose fibrotic tissues; f+ca: dense fibrous collagenous network containing calcified necrotic remnants of cardiomyocytes. Size bar: 50 µm in c (same magnification in a, b, d-f).

Figure S4. LC3 protein expression is localized in cardiomyocytes and in the cell infiltrate surrounding necrotic cardiomyocytes of *Dsg2^{cKO}* mice during structural disease onset

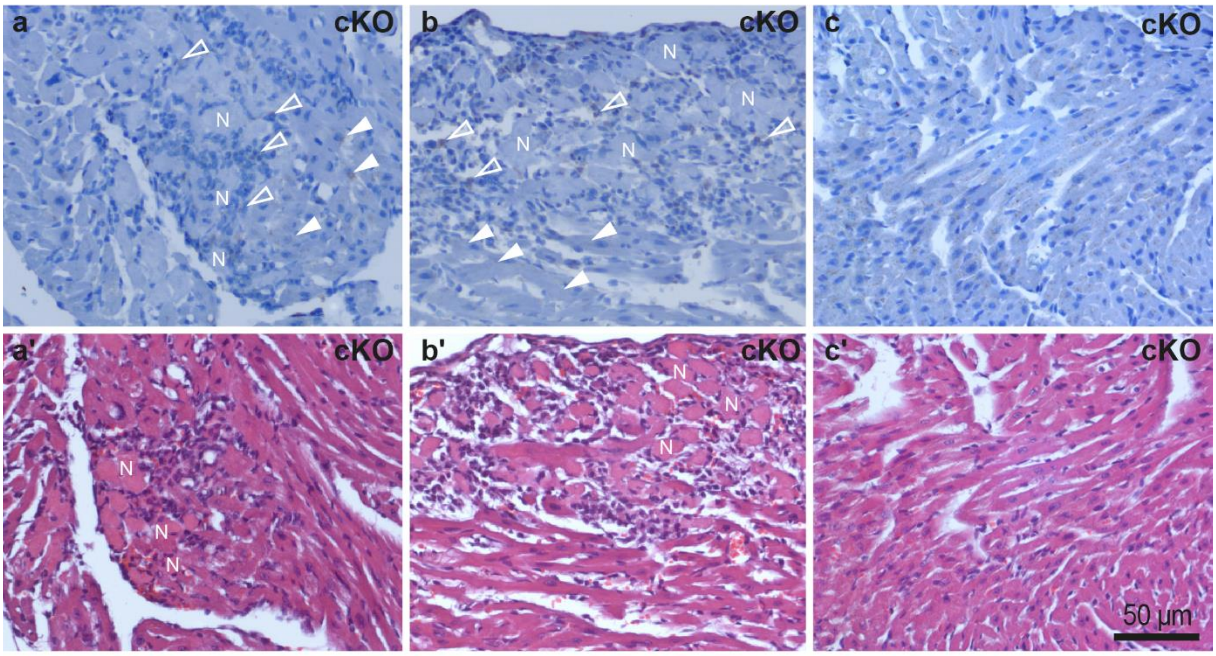

The sections were taken from 18 day-old *Dsg2^{cKO}* mice. (a-c) depict LC3 immunostaining (brown) with corresponding serial hematoxylin/eosin stained sections in (a'-c'). Necrotic cardiomyocytes are marked with "N". (a) shows a papillary muscle, (b, c) show the right ventricle. LC3-positive cardiomyocytes are marked by white arrowheads and LC3-positive immune or interstitial cells are delineated by open arrowheads in (a, b). The LC3 staining intensity of lesion-near cardiomyocytes (a, b) is similar to that detected in the healthy-appearing remote mutant myocardium (c).

Figure S5. SQSTM1/p62 immunohistochemistry in healthy control animal

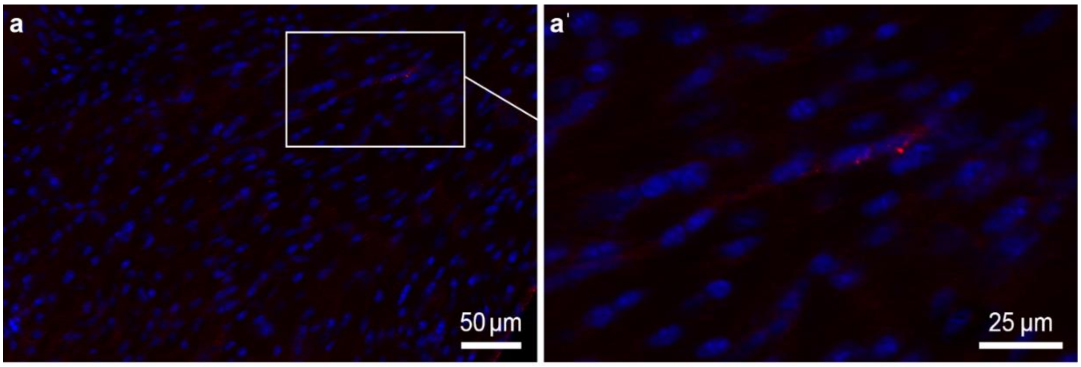

In healthy control mice SQSTM1/p62 positive granules (red) were only rarely detectable. Nuclei are labeled with Hoechst 33342 dye (blue).

Figure S6. In situ hybridization controls show mRNA preservation and detection specificity in 4 week-old hearts

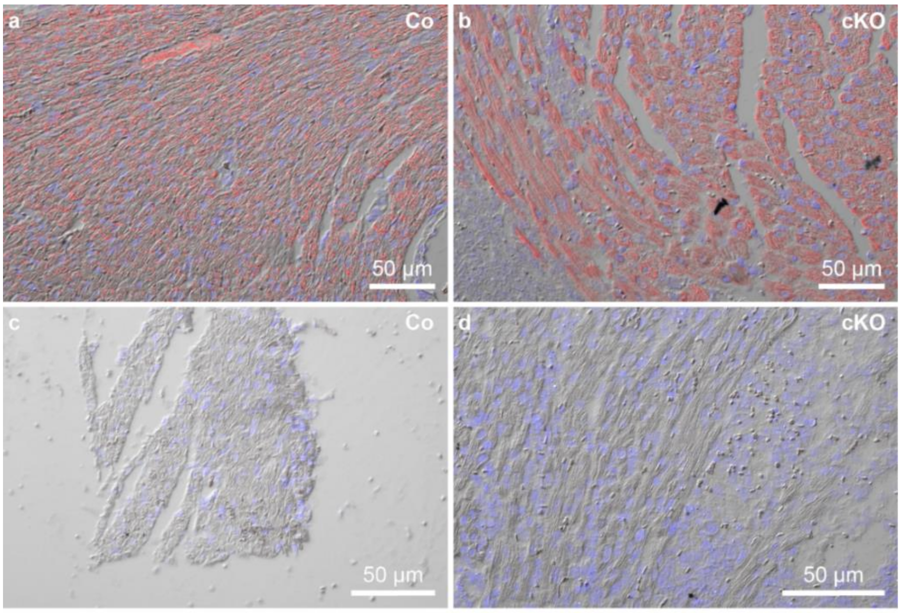

(a, b) show micrographs detecting Actc1 mRNA by in situ hybridization (red signal) in control *Dsg2*$^{flox(E4-5)}$ (Co) and *Dsg2*^{cKO} (cKO) hearts. (c, d) show the corresponding negative controls, in which the specific hybridization probe was omitted. Nuclei are labeled with Hoechst 33342 dye (blue).

Figure S7. In situ hybridization controls show mRNA preservation and detection specificity in 30 week-old hearts

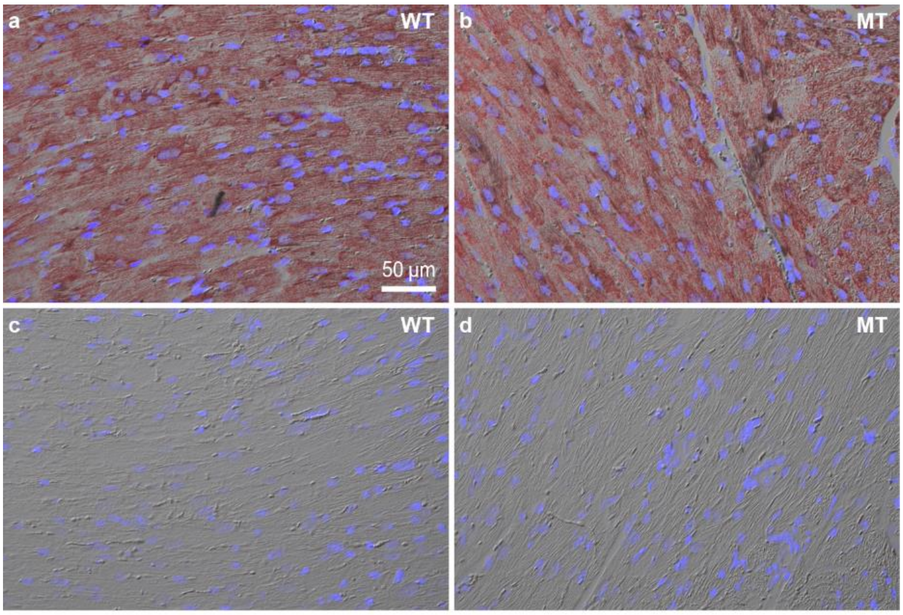

(a, b) show micrographs detecting Actc1 mRNA by in situ hybridization (red signal) in control *Dsg2*^{WT} (WT) and *Dsg2*^{MT} (MT) hearts. (c, d) show the corresponding negative controls, in which the specific hybridization probe was omitted. Nuclei are labeled with Hoechst 33342 dye (blue). Size bar, 50 µm in a (same magnification in b-d).

Figure S8. In situ hybridization controls show mRNA preservation and detection specificity in 1 year-old hearts

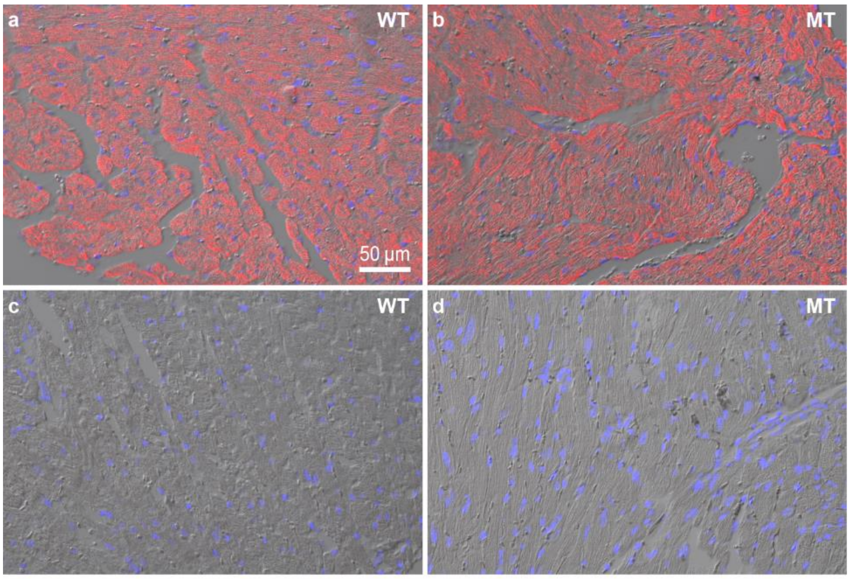

(a, b) show micrographs detecting Actc1 mRNA by in situ hybridization (red signal) in control *Dsg2*^{WT} (WT) and *Dsg2*^{MT} (MT) hearts. (c, d) show the corresponding negative controls, in which the specific hybridization probe was omitted. Nuclei are labeled with Hoechst 33342 dye (blue). Size bar, 50 µm in a (same magnification in b-d).

Figure S9. The number of autophagic vacuoles is not increased in 12 week-old *Dsg2^{MT}* mice

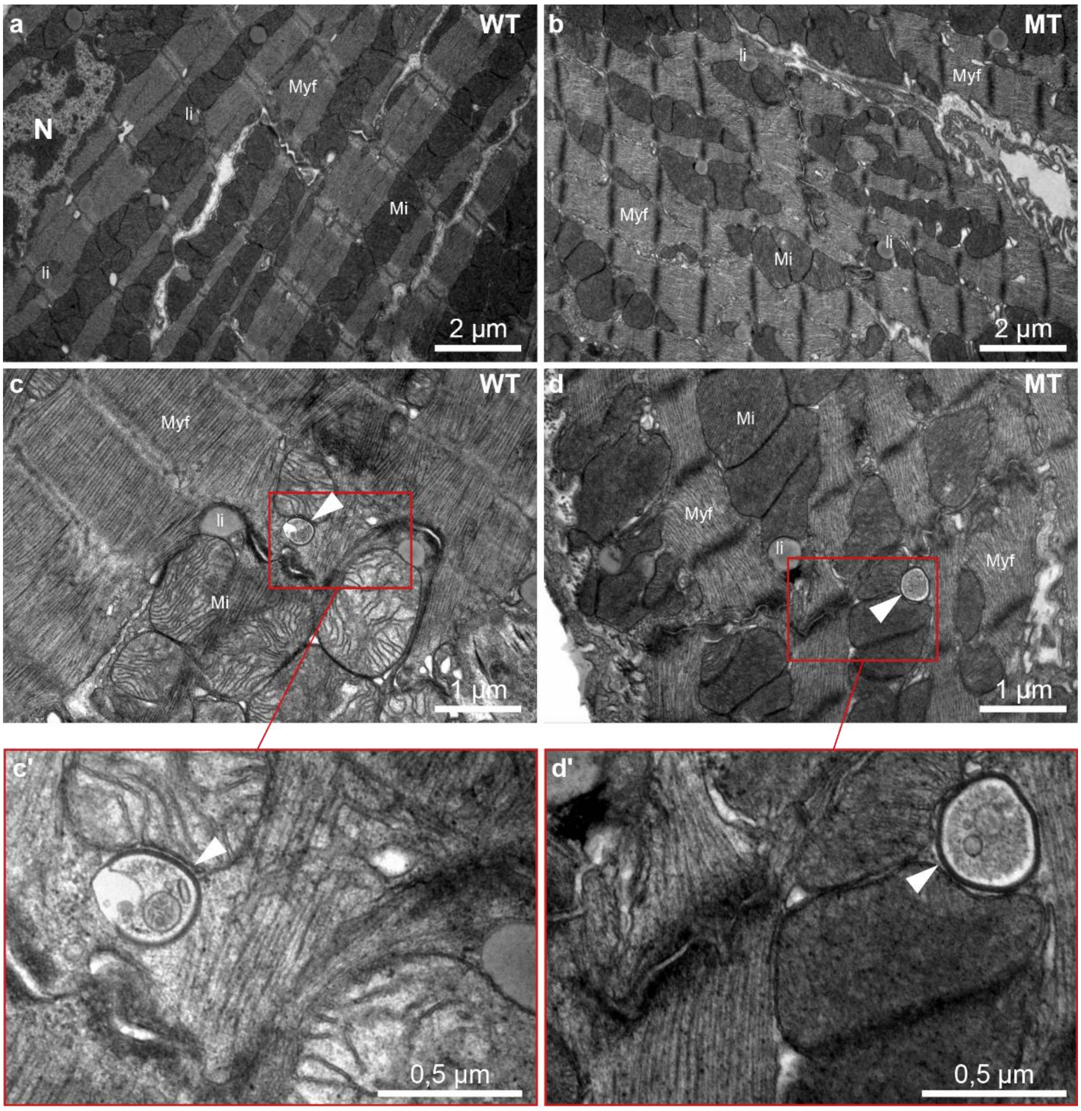

The transmission electron micrographs were recorded in sections of 12 week-old wild-type control (WT; a, c, c') and *Dsg2^{MT}* mice (MT; b, d, d'). While large parts of wild-type and *Dsg2^{MT}* myocardium do not show any morphological signs of autophagy (a and b), single autophagosomes can be found in both groups (white arrowheads in c and d). c' and d' are magnifications of the boxed areas in c and d, respectively. li, lipid droplet; Mi, normal mitochondrion; Myf, myofibrils; N, nucleus.

Figure S10. Chop mRNA expression is increased in single cardiomyocytes in *Dsg2*-mutant hearts

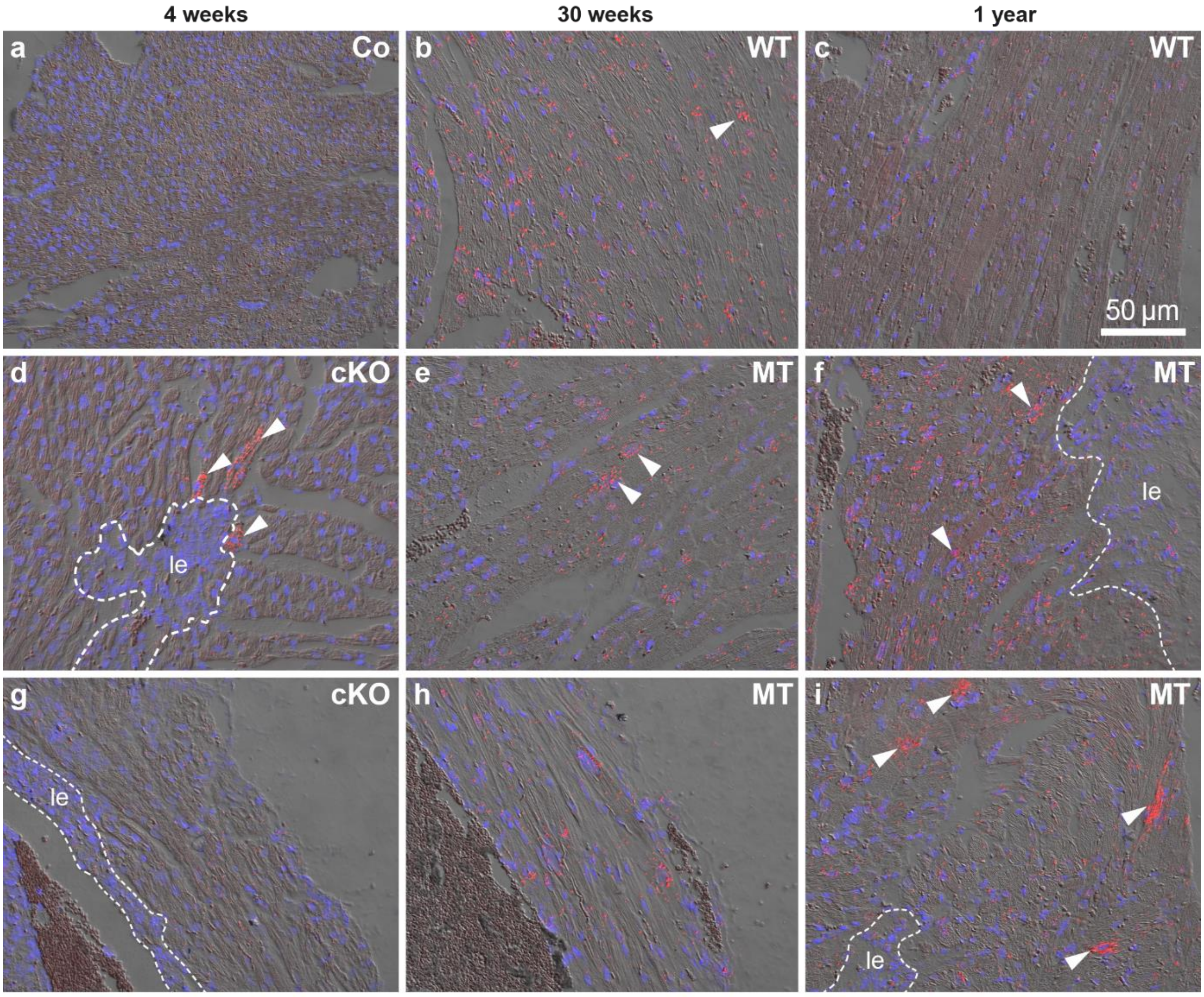

The photomicrographs show the results of in situ hybridization experiments detecting Chop mRNA in the hearts of 4 week-old *Dsg2*$^{flox(E4\text{-}5)}$ (Co) and *Dsg2*^{cKO} (cKO) mice (a, d, g), 30 week-old wild-type (b) and *Dsg2*^{MT} (MT) mice (e, h), and 1 year-old wild-type (c) and *Dsg2*^{MT} (MT) mice (f, i). Note, that Chop mRNA overexpressing cardiomyocytes (arrowheads in d) are located next to a lesion (le, demarcated by dashed line) in the left ventricle at 4 weeks whereas no such cells are detectable in the right ventricle (g). In 30 week-old mutants, some cardiomyocytes with elevated Chop mRNA expression are detected in the left (e) and thinned right ventricle (h). In 1 year-old *Dsg2*^{MT} mutant mice cardiomyocytes with elevated Chop mRNA expression (arrowheads) are seen in close proximity to established lesions (f, i). Size bar: 50 µm in c (same magnification in a, b, d-i). Nuclei are labeled with Hoechst 33342 dye (blue).

Figure S11. Apoptotic cleaved caspase 3-positive cells are not increased in perilesional *Dsg2*-mutant myocardium

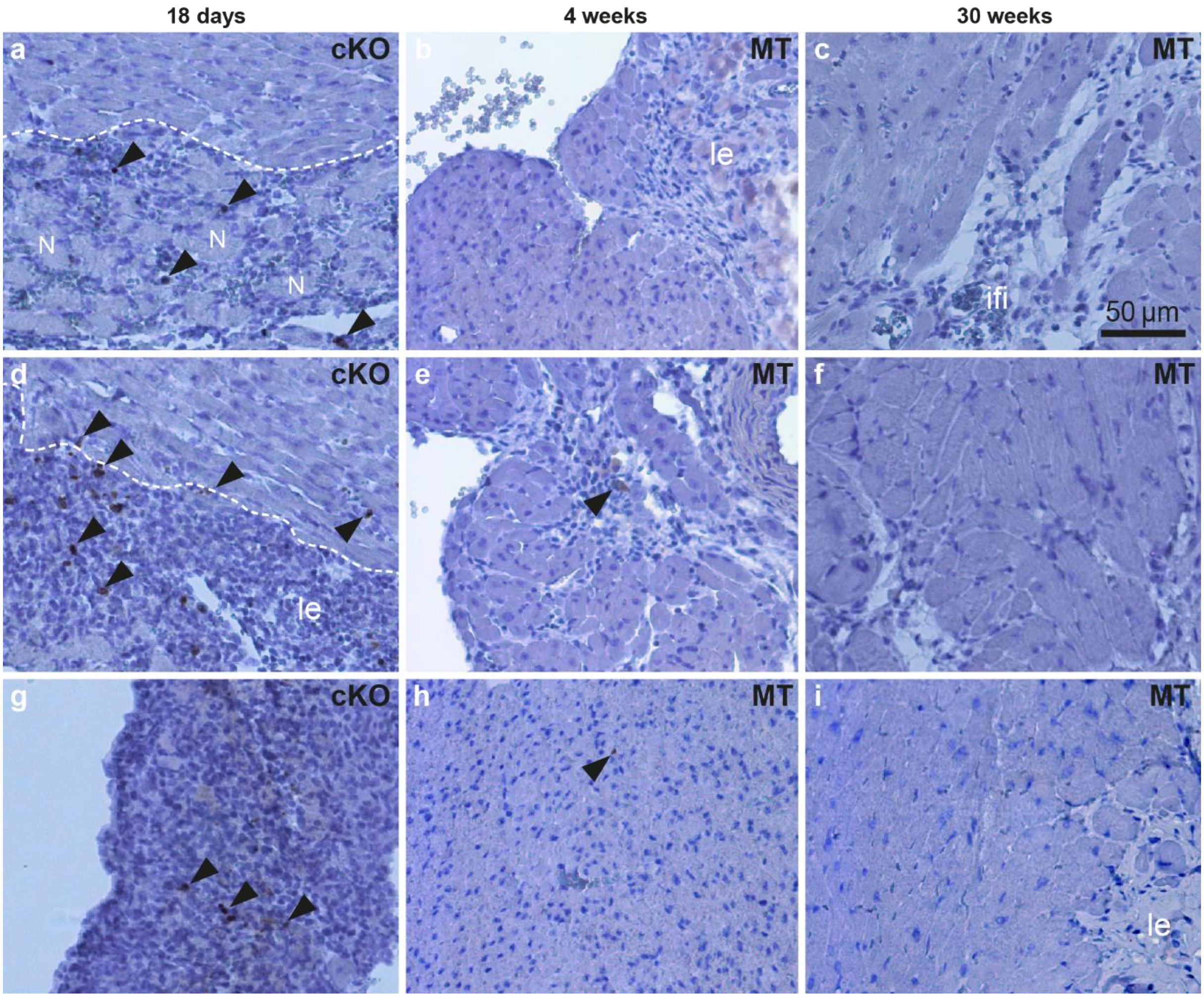

The photomicrographs present cleaved caspase 3 (CC3) immunohistochemistry in heart sections of $Dsg2^{cKO}$ mice (cKO; a, d, g) at 18 days and $Dsg2^{MT}$ (MT) mice at 4 and 30 weeks (b, c, e, f, h, i). Numerous CC3-positive cells are detected (arrowheads) in immune cell infiltrates surrounding necrotic cardiomyocytes (N; a), in lesions (le; demarcated by dashed line; d) or throughout the complete lesioned right ventricular wall (g) of 18 day-old $Dsg2^{cKO}$ *hearts*. In the immature lesions of 4 week-old $Dsg2^{MT}$ mice (b, e) and in the normal appearing remote myocardium (h) only single CC3-positive non-cardiomyocytes are detected. The 30 week old $Dsg2^{MT}$ myocardium (c, f, i) contains no CC3-positive cardiomyocytes (although 1-2 positive cells are detectable per section). ifi, interstitial fibrosis. Size bar: 50 µm in c (same magnification in a, b, d-i).
